# Creation and Implementation of an EMS Elective for Final-Year Medical Students: A 5-year Evaluation

**DOI:** 10.5811/westjem.35419

**Published:** 2025-02-28

**Authors:** Edder Peralta, Christopher Evers, Toniann Gonell, Megan Hodges, David Cohen, Lauren M. Maloney

**Affiliations:** Stony Brook Medicine, Department of Emergency Medical Services, Stony Brook, New York

## Abstract

**Introduction:**

Emergency medical services (EMS) professionals interact with nearly every type of physician and are key stakeholders across the healthcare spectrum. However, no formal national recommendations exist for medical student education about EMS. When looking for institution-level resources to assist in writing the educational objectives and curricular content for an EMS elective for medical students, limited examples are available for guidance. We designed, implemented, and evaluated a two-week EMS elective for final-year medical students. A pragmatic description of how to create an EMS elective is detailed.

**Methods:**

The EMS elective involves an introductory session, an operational orientation, and six ambulance shifts. Self-directed activities and checklists encourage interdisciplinary learning between calls. Additionally, students deliver a case presentation including an example for improved interdisciplinary communication. Before and after the elective, a voluntary anonymous survey is distributed, in addition to a formal standard course evaluation.

**Results:**

From 2017–2022, 37 students participated in the elective. Thirty-four (92%) submitted the pre-elective survey, and 21 (57%) submitted the post-elective survey. Mann-Whitney U testing suggested an improved understanding of the capabilities of different EMS practitioner levels and of the different types of medical oversight after the elective (median pre=60%, median post=90%, U=118, P<0.001). Qualitatively, students described their experiences as “practical,” “hands-on,” and “eye-opening.”

**Conclusion:**

An EMS elective using andragogy and intentional interdisciplinary communication seems useful in facilitating improved understanding of the fundamentals of EMS practice for final-year medical students.

## BACKGROUND

Emergency medical services (EMS) professionals, via their current roles within 9-1-1 emergency response, critical care interfacility transfers, and prescheduled movement of medically complicated patients in the outpatient setting, interact with nearly every physician specialty. With advances in mobile-integrated health and community paramedicine, EMS professionals are anticipated to become even more integrated within the healthcare spectrum, thereby strengthening these physician-EMS interactions.^1^

Given the importance of future physicians’ understanding of the fundamentals of contemporary EMS practice, regardless of their specialty or practice setting (eg, inpatient, outpatient, telehealth), offering medical students the opportunity to work directly with EMS professionals would seem to be an invaluable experience. However, despite ubiquitous interactions between EMS professionals and physicians, no formal national recommendations currently exist about how to introduce medical students to EMS systems and the crucial role EMS plays within the healthcare system. National educational guidance related to educating physicians about EMS first appears at the resident physician level with the Accreditation Council for Graduate Medical Education requiring that emergency medicine (EM) residents “have experience in emergency medical services… includ[ing] ground unit runs and should include direct medical oversight.”^2^ A model EMS curriculum for EM residents has been proposed by the Society for Academic Emergency Medicine; however, it dates back to 1996.^3^

When looking for institution-level resources to assist in writing the educational objectives and curricular content for an EMS elective for medical students, limited examples are available for guidance. Superficial course descriptions can be found on individual medical school websites via internet search; however, concrete details about scheduling, learning materials, and assessment strategies are generally not readily available to the public. Although the value of medical student exposure to EMS through emergency medical responder courses, emergency medical technician courses, or advanced elective opportunities, have been referenced in retrievable literature, these opportunities are often limited to medical schools affiliated with university hospitals that have strong, well-established connections with EMS agencies.^4^–^10^ For medical schools without robust pre-existing relationships with surrounding EMS agencies, descriptions about how to create and implement an EMS elective by way of offering ready-to-use resources and including pragmatic feedback on how to overcome operational challenges inherent to EMS to provide optimal medical student learning experiences, are nearly non-existent.

## OBJECTIVES

Our goal was to design, implement, evaluate, and disseminate a model curriculum for a two-week EMS elective for final-year medical students who have already completed their EM clerkship experience.

## CURRICULAR DESIGN

### Study Design

Prospective, observational data collection from the medical students taking the course was planned for during the initial development of the EMS elective to allow for dynamic curriculum changes to be made in response to student and faculty feedback. The proposed data collection plan was reviewed and approved by the Stony Brook University institutional review board.

### Study Setting & Participants

The Renaissance School of Medicine (RSOM) at Stony Brook University is an allopathic state medical school with approximately 130 medical students per graduating class, located on Long Island. The primary clinical site for RSOM is Stony Brook University Hospital (SBUH), a suburban, academic, Level I trauma and tertiary care center with an annual emergency department census of approximately 110,000 patients. Stony Brook University Hospital Emergency Medical Services (SBUH EMS) is the hospital-based EMS agency, which employs approximately 100 paramedics and 50 emergency medical technicians. SBUH EMS has an annual call volume of approximately 15,000 requests for service, which is comprised of 9-1-1 and interfacility responses. The 9-1-1 response vehicles include multiple ground ambulances, mobile stroke units, and rotor-wing air ambulances.

**Population Health Research Capsule**

What do we already know about this issue?

*Although emergency medical service (EMS) professionals interact with nearly every physician specialty, no formal recommendations exist for what medical students should learn about EMS systems.*

What was the research question?

*We designed, implemented, and evaluated a curriculum for an EMS elective for final-year medical students.*

What was the major finding of the study?

*We found an improved understanding of EMS practitioner levels and medical oversight (pre score=60%, post score=90%, U=118, P<0.001).*

How does this improve population health?

*An EMS elective using andragogy and intentional interdisciplinary communication can improve medical student’s understanding of the fundamentals of EMS practice.*

### Curriculum Developmental Process

The overarching goals of the EMS elective were to provide medical students with high-yield educational content that would be beneficial regardless of their future specialty, as well as to encourage a two-way learning opportunity between EMS professionals and medical students. Medical students may lament that at the end of their medical school experience, while they may feel more knowledgeable about anatomy, physiology, pathology, and the healthcare system, their understanding of practical medicine, such as how to turn on an oxygen tank, assemble a nebulizer mask, or apply 12-lead electrocardiogram electrodes, may be lacking. An EMS elective offers the opportunity for EMS professionals to showcase their unique skillset for the future physicians with whom they will be interacting, while medical students are simultaneously encouraged to share with EMS professionals their newly learned understanding of anatomy, physiology, and pathology.

The initial curriculum for the elective was assembled based on the experience of the course director, LMM, a critical care paramedic and nationally certified EMS educator who went on to become a double board-certified emergency physician and EMS physician. To guide EMS-specific medical knowledge, the following course objectives were developed:

1. Describe an understanding of the components of EMS including educational requirements and scope of practice for different levels of EMS professionals, operations of the local EMS system, fundamentals of communication, and documentation requirements;
2. Appreciate the time, safety, personnel, and equipment constraints of prehospital care;
3. Discuss the different forms of medical direction;
4. Explain the importance of timely, precise interdisciplinary communication and safe patient-hand-off practices.

The educational activities (Table 1) composing the EMS elective were developed based on andragogy and incorporated self-directed and experiential learning theories (Appendix A or from the authors upon request). In addition to course objectives, the educational activities are linked with the Association of American Medical Colleges’ Entrustable Professional Activities and Physician Competencies (Table 2).^11^–^12^

### Course Structure

The EMS elective, offered bimonthly, is two weeks long and has 2–3 seats available per iteration. The elective begins with a one-hour operational orientation over Zoom. The course director reviews the course syllabus, discusses safe attire and equipment expectations, and orients the students to their schedules and locations of ambulances. Students then asynchronously watch a one-hour long introductory lecture pre-recorded by the course director. Clinically, medical students are scheduled for three 9-1-1 ambulance shifts, two critical care interfacility transport shifts, and one mobile stroke unit shift, totaling 64 clinical hours. (If critical care interfacility transport shifts and/or mobile stroke unit shifts are not available to a medical school, having all ride-along shifts on 9-1-1 ambulances would likely be an equally meaningful clinical experience.)

A student skill-tracking form is sent along with an introduction e-mail to orient paramedic preceptors to meaningful skills and valuable educational opportunities specific to medical students, as SBUH EMS paramedic preceptors often have multiple learner-types regularly joining them for ride-alongs. During the ride-along shifts, medical students complete the student skill-tracking form and a patient encounter log. During down time between calls, medical students complete an open-book protocol, review scholarly articles related to EMS, and complete a worksheet for each article.

On the final day, each medical student delivers a case presentation, which includes a protocol review, a reference to a peer-reviewed article, and a description of one opportunity for improved interdisciplinary communication. After the student submits their completed educational activities, the annotated answers to the open-book protocol exam and the scholarly article worksheets are sent to the students to ensure their correct understanding of the content.

Before and after the elective, a voluntary, anonymous survey containing demographic, opinion, and knowledge-based questions is distributed, which was reviewed by the SBU IRB. The post-elective survey is not sent until after the students’ final grades are posted. These surveys were initially handed to students in paper form; however, during the pandemic, they were moved to an online format. Additionally, students complete formal standard course evaluations administered by the RSOM.

### Course Implementation

In keeping with the axiom that “if you’ve seen one EMS system, you’ve seen one EMS system,” we offer some pragmatic guidance for medical school faculty who may be interested in creating an EMS elective. When sending medical students for ambulance ride-along time, it is important to consider whether an affiliation agreement is needed to ensure appropriate medical malpractice coverage is in place, as well as to confirm what procedural skills medical students are allowed to participate in (ie, endotracheal intubation, intraosseous needle placement). Once the legal coverage is in place, it would be beneficial for the course director to establish a single point of contact within the EMS agency. This individual acts as the liaison with the department, helps orchestrate the scheduling, confirm staffing (location, shift times), troubleshoots real-time problems (eg, if a medical student were to arrive late due to car trouble and needs to rendezvous with the ambulance), and acts as a point of contact in the event of a workplace injury, occupational exposure, or motor vehicle collision.

It is also important to establish what equipment the medical student is expected to bring with them during the ride-alongs. For example, do the students need to bring their own high-visibility safety vest, personal protective equipment, helmet, and turnout gear, or is that provided by the EMS agency? Special attention must be paid to what clothing the medical students wear (closed-toe, supportive shoes are a must), and specific guidance should be given regarding wearing identification badges and branded clothing.

For course directors who may feel they do not have a strong background in EMS, it would be beneficial to consider involving a senior paramedic or supervisor when delivering the introductory lecture or operational orientation. Introductory lecture content material is well covered in general EM textbooks such as Tintanelli’s or Rosen’s.^13^,^14^ For a deeper dive into content, textbooks used by most EMS fellowships could be considered.^15^

## IMPACT/EFFECTIVENESS

Before and after the elective, we distributed a voluntary, anonymous survey (reviewed by the SBU IRB) containing demographic, opinion, and knowledge-based questions. Trends were elicited using descriptive statistics and Mann-Whitney U testing. Recurring themes were elicited from the open-response questions. After completing the elective, students are asked to complete a standardized formal course evaluation administered electronically by the RSOM. These results are provided as an anonymous, aggregated set annually to the course director.

From 2017–2022, 37 medical students participated in the elective, five of whom reported prior EMS experience. Anticipated residencies predominantly included EM (18), anesthesiology (5), and internal medicine (4). Other anticipated residencies included pediatrics, obstetrics and gynecology, neurology, neurosurgery, urology, and psychiatry.

Students averaged 12 patient encounters (range 5–21) during the elective. All students completed the skill-tracking form and delivered a case presentation at the end of the elective, demonstrating an understanding of the prehospital patient course, the protocols involved, and the initial workup in the emergency department. Each student recognized, described, and put into the context of patient safety and systems-based practice at least one opportunity for improved communication between medical professionals, and interpreted the call in the context of a scholarly article.

Thirty-four students (92%) submitted a pre-elective survey, and 21 (57%) submitted a post-elective survey; not all opinion prompts on the surveys were answered (Table 3). Mann-Whitney U testing suggested a significantly improved understanding of the capabilities of different EMS clinician levels and of the different types of medical oversight after the elective (median pre-elective score = 60%, median post-elective score = 90%, U = 118, *P*<0.001). Qualitatively, students repeatedly used the terms “practical,” “involved,” “included,” “hands-on,” and “eye-opening” when describing their experiences.

Over the five years, 33 of 37 medical students completed the standardized formal course evaluation administered electronically by the RSOM (Table 4).

An EMS elective based on andragogy and emphasizing intentional interdisciplinary communication, while being cognizant of faculty responsibilities, seems useful in facilitating an improved understanding of the fundamentals of EMS practice for final-year medical students and attracts students beyond those interested in emergency medicine. Quantitatively, students demonstrated an improved understanding of fundamental components of EMS, such as medical direction and scope of practice for different certification levels (Table 5).

Qualitatively, students were immersed in a world distinct from what they are used to in-hospital. Many of the procedures are novel, and those that are familiar now have unusual nuances, such as being performed in patients’ homes. This affords the opportunity to obliterate perceptions of medical hierarchy as medical students and EMS professionals become equal partners in the exchange of information. Medical students offer knowledge from their clerkship activities. In return, the opportunity for EMS professionals to impart their unique skill-set to individuals who will become the physicians receiving their patients builds camaraderie and mutual respect. This value in recognizing complementary strengths is especially evident with the student skill-tracking form as it offers medical students an opportunity in a low-stakes environment to ask what they may perceive to be “silly” questions, such as how to apply oxygen to a patient or even confirm that an oxygen tank does indeed have oxygen in it.

With this interdisciplinary immersion also comes the direct opportunity to experience how crucial effective, timely communication is and the consequences of poor communication. This is intentionally brought to their conscious thought when reading a mandatory scholarly article about handoffs and again when they deliver a case presentation at the end of the elective. Collectively, these experiences seem to have positively impacted medical student awareness of professionalism, interpersonal and communication skills, and systems-based practice. Finally, the open-ended questions on the post-elective survey encourages students to reflect on their role as both givers and receivers of information as they continue their career-long endeavor of practice-based learning and improvement.

In response to student feedback, the ratios of different ride-along types have been adjusted. Before the COVID-19 pandemic, students spent more time on interfacility, critical care transport ambulances, spent a shift in the medical control office for the county, and had the opportunity to ride in an SUV first-responder fly car. Due to physical distancing requirements when the elective resumed following the peak of the pandemic, medical control and the fly cars were removed; prior student feedback also suggested these were the least productive of their experiences. Additionally, the department has seen a change in types of requests for service, with more call volume coming from 9-1-1 ambulances and less call volume coming for critical care interfacility transportation. Therefore, students now spend more time on 9-1-1 ambulances and the mobile stroke unit and less time with the critical care transport teams.

Acknowledging medical student expectations of the course load of an elective, we have considered adding an introduction to disaster management and incident command via FEMA IS-100.C. We have also considered adding interdisciplinary simulation cases to reduce experiential gaps caused by the unpredictable nature of 9-1-1 calls. It also deserves specific mention that the engagement and interest of paramedic preceptors is crucial for students’ positive experiences. Timely, appreciative feedback is provided to preceptors with inclusion of agency leadership, and quotes from students are included in annual EMS weekly emails to the department in recognition of their efforts.

## LIMITATIONS

The most significant limitation of this study is the quantitative feedback that was received. After the elective resumed following the initial COVID-19 pandemic peak, the voluntary, anonymous pre- and post-course evaluations were moved to an online format, which reduced response rate. Despite specialty unit involvement, the content and focus of the elective as far as skills, knowledge, and interpersonal skills have stayed true to the central tenants of EMS medicine. Thus, we believe our educational resources can be implemented in other settings with a reasonable degree of adaptation to meet their local needs.

## CONCLUSION

An EMS elective using andragogy and intentional interdisciplinary communication seems useful in facilitating an improved understanding of the fundamentals of EMS practice for final-year medical students. Given limited, if any, guidance on how to educate future physicians about the most crucial aspects of emergency medical services, we offer a pragmatic description about how to start an EMS elective, including course objectives tied to national medical school education competencies, sample syllabi and educational activity templates, and recommendations on how to overcome operational challenges inherent to EMS such as scheduling, liability, and communication, to encourage an optimal medical student learning experience.

## Supplementary Information



## Figures and Tables

**Table 1:** Components of two-week emergency medical services elective and the course objectives they meet.

Educational activity	Description	Course objective
Introductory lecture	One-hour, prerecorded, asynchronous, instructor-centered didactic session. Lecture begins with discussion of the history of EMS and then describes the fundamentals of modern EMS systems. A progressive disclosure case presentation takes medical students into the mind of a paramedic regarding scene safety, crew resource management, environmental and operational considerations, and spatiotemporal awareness.	1, 3
Ambulance ride-along	Experiential learning opportunity.	1–4
Student skill-tracking form	List of items to discuss, review, or perform during the ride-along time.	1, 2, 4
Patient encounter log	Form to track patient encounters to confirm robust clinical exposure.	1, 2, 4
Open-book protocol exam	Self-directed learning opportunity during down time on ride-alongs to become familiar with EMS medicine. A series of multiple-choice questions guide the student through the protocols, highlighting protocol structure, unique aspects of EMS medicine, and the scope of practice of different EMS professionals.	1 – 4
Scholarly articles	Self-directed learning opportunity during down time on ride-alongs to review EMS-related literature. One article on EMS- to-emergency-department hand-offs is mandatory; the student chooses two of the additional four articles provided to read based upon their interests. Students complete a corresponding worksheet for each article.	1, 4
Case presentation checklist	A case presentation guided by a checklist that includes a protocol review, a reference to a peer-reviewed article, and a description of at least one opportunity for improved interdisciplinary communication.	1 – 4

*EMS*, emergency medical services.

**Table 2:** A description of the AAMC* Entrustable Professional Activities and Physician Competencies linked to the educational activities that comprise the elective.

Entrustable Professional Activity^11^	Physician Competency reference set^12^	Educational activity
1. Gather a history and perform a physical examination.	PC2, ICS1, ICS7, P1, P3, P5, KP1	Patient encounter log Case presentation checklist
2. Prioritize a differential diagnosis following a clinical encounter.	PC2, KP3, ICS2, ICS3	Case presentation checklist
6. Provide an oral presentation of a clinical encounter.	PC2, PC3, PC4, PC5, PC6, ICS2, ICS3, PBLI1	Case presentation checklist
7. Form clinical questions and retrieve evidence to advance patient care.	KP3, PBLI1, PBLI3, PBLI6	Case presentation checklist
8. Give or receive a patient handover to transition care responsibly.	ICS2, ICS3, P3	Case presentation checklist Student skill-tracking form
9. Collaborate as a member of an interprofessional team.	IPC1, IPC2, IPC3, IPC4, SBP1, ICS2, ICS3, ICS7, PBLI8	Patient encounter log. Case presentation checklist Student skill-tracking form
10. Recognize a patient requiring urgent or emergent care and initiate evaluation and management.	PC1, PC2, PC4, PC5, PC6, IPC4, ICS2, ICS3	Case presentation checklist Open-book protocol exam
12. Perform general procedures of a physician.	PC1	Student skill-tracking form
13. Identify system failures and contribute to a culture of safety and improvement.	KP1, ICS2, SBP4, SPB5	Case presentation checklist

* *AAMC*, Association of American Medical Colleges.

**Table 3:** Medical student responses to post-elective anonymous course evaluations.

Prompt	Responses
Is there anything that stood out in your memory about the elective (e.g., an experience, an interaction)?	- During one of the 9-1-1 calls I was a part of, I remember how quickly but methodically the medic worked through all the tasks we needed to complete. One of my primary goals at the end of my training in EM is to be able to manage a super-unstable patient that well. - The rapport that the paramedics built with the patient during the transport - How integral a role EMS plays in patient care. I think this is unappreciated by medical professionals. - The amazing MSU system- every part of it. The organization, the skills, the teamwork, the technology, and the care.
If you were asked to give a lecture to a group of EMTs or paramedics, what topics would you speak about?	- The importance of communication - Cultural competency when taking care of LGBTQ+ patients - Managing a laboring patient - Tips for caring for newborns and young pediatric patients
If a paramedic were to give a lecture to a group of medical students, what topics would you like to hear about?	- Scene management - Some of the experiences prehospital providers go through on scene that hospital providers never see - Practical medicine (drug math, physically moving patients) - Clinical pearls about running codes in unconventional or uncontrolled settings - Thought process during initial interventions - Thought process during MCIs - How their HPI and exam can be limited due to patients’ living conditions, initial location in the house, physical obstacles; and how to overcome those challenges

*EMS*, emergency medical services; *EMT*, emergency medical technician; *MCI*, mass casualty incident; *HPI*, history of present illness; *MSU*, mobile stroke unit; *EM*, emergency medicine; *LGBTQ*, Lesbian, Gay, Bi-sexual, Transgender, Queer, or Questioning.

**Table 4:** Aggregate results of five years of medical student responses to the standardized formal course evaluation administered electronically. Of the 37 students who took the elective, 33 completed the evaluation.

Prompt	Strongly disagree n (%)	Disagree n (%)	Indifferent n (%)	Agree n (%)	Strongly agree n (%)
This course had clear learning objectives.	1 (3%)			4 (12%)	28 (85%)
This course met its stated objectives.	1 (3%)			4 (12%)	28 (85%)
The teaching methods were appropriate for the stated objectives.	1 (3%)			4 (12%)	28 (85%)
The evaluation methods were clear.	1 (3%)			5 (15%)	27 (82%)
The evaluation methods were applied consistently and fairly.	1 (3%)			5 (15%)	27 (82%)
The course content was relevant and of sufficient detail.	1 (3%)			4 (12%)	28 (85%)
Adequate time was provided in this course to meet the learning objectives.	1 (3%)			5 (15%)	27 (82%)
There was good integration of basic science and clinical correlates in this course.	1 (3%)			5 (15%)	27 (82%)
The learning materials in this course were appropriate.	1 (3%)			5 (15%)	27 (82%)
Overall, this course was well structured.	1 (3%)			5 (15%)	27 (82%)
Overall, I am very satisfied with this course.	1 (3%)			4 (12%)	28 (85%)
I would recommend this elective to future students.	1 (3%)			4 (12%)	28 (85%)

Additional comments:
-Excellent opportunity for emergency medicine candidates and other students alike to gain exposure to pre-hospital medicine and what happens before patients get to the front door of the hospital. Would highly recommend this course as an experience for any medical student. -Well organized, with the schedule of shifts sent to us in advance. Also, great opportunity to learn about the role of EMS, and the relationship between physicians and EMS. -It was so much fun and such a great learning opportunity to get to work hands on with patients in highly acute situations. You learn so much about the prehospital and transport world that’ll benefit me for years to come. -This is a great course. It’s very helpful to provide a better understanding of how EMS works and the challenges they face daily. -This elective was one of the best structured with appropriate checklists and well thought-out learning points. Everyone involved was aware of incoming students and excited to teach. -Working alongside extremely knowledgeable and friendly prehospital staff and learning practical life-saving skills.

**Table 5:** Aggregate results of five years of medical student responses to medical knowledge questions on pre- and post-course surveys as analyzed by Mann-Whitney U testing.

Question	Students Answering Correctly n (%)		U value, P-value

	Pre n = 34	Post n = 21	
In NY, the highest level of 9-1-1 prehospital care is provided by: (paramedic)	16(47%)	16(76%)	U=253, P=0.04
In NY, EMT-Basics are allowed to administer all the following medications except: (morphine)	27(79%)	13(62%)	U=295, P=0.16
In NY, EMT-Basics are allowed to perform all the following skills except: (IV insertion)	21(62%)	17(81%)	U=289, P=0.14
In Suffolk County, [9-1-1] paramedics are allowed to administer all the following medications except: (propofol)	21(62%)	19(90%)	U=255, P=0.02
In Suffolk County, paramedics are allowed to perform all the following skills except: (pericardiocentesis)	31(91%)	17(81%)	U=321, P=0.27
Prehospital care professionals work under the medical license of: (their medical director)	28(82%)	21(100%)	U=294, P=0.04
In NY, a paramedic’s initial education is at least how many hours: (1,000)	18(53%)	20(95%)	U=206, P=0.001
In NY, an EMT-Basic’s initial education is at least how many hours: (150)	16(47%)	14(67%)	U=287, P=0.16
When a paramedic provides an intervention to a patient without calling a physician, they are using: (standing orders)	12(35%)	17(81%)	U=194, P=0.001
When a paramedic is on scene and wants to perform an intervention not specifically mentioned in their protocols, they must: (call the medical control physician)	20(59%)	19(90%)	U=244, P=0.01
