# Supplementary material for: Creation and Implementation of an EMS Elective for Final-Year Medical Students: A 5-year Evaluation
Source: West J Emerg Med. 2025 Feb 28;26(3):556–63. doi: 10.5811/westjem.35419 (PMC12208031; doi:10.5811/westjem.35419)
Supplement: Supplementary file 1 [file wjem-26-556-s001.pdf]

Appendix A: Course Syllabus

Course Title: Prehospital Emergency Medicine Elective

Course Director:

Course Information:

Duration	2 weeks
Students	2
Objectives	- Develop an understanding of the components of prehospital emergency medicine including:<br>o Educational requirements and scope of practice for different levels of EMS providers<br>o Local EMS system operations<br>o Fundamentals of communication<br>o Documentation requirements<br>- Develop an awareness of the time, safety, personnel, and equipment constraints of prehospital emergency care<br>- Develop a basic understanding of the prehospital management of medical and trauma patients<br>- Develop an awareness of the different forms of prehospital medical direction (offline and online) as well as their pitfalls
Prerequisites	- Phase III student<br>- Successful completion of an Emergency Medicine Clerkship
Description	This two-week elective is designed to expose medical students to the principles of prehospital emergency medicine.<br><br>On the first day of the elective, students will receive didactic instruction to provide a foundational understanding of the history of emergency medical services (EMS), local and national EMS system structure, and the role of the Emergency Medicine Physician within the EMS system.<br><br>Clinical experiences will occur via ride-alongs with EMS. Students will spend three shifts as a ride along with EMS on a 911 ambulance and one shift as a ride along with a Mobile Stroke Unit. Additionally, students will spend two shifts as a ride along with EMS paramedics during interfacility transports.

Appendix A: Course Syllabus

	During clinical shifts with EMS, students are expected to complete a Patient Encounter Log and discuss/perform all the skills listed on the Student Skill Tracking Form. Additionally, students will complete an open book protocol quiz intended to familiarize students with EMS protocol structure, content, and considerations when providing medical care that are unique to EMS.<br><br>Additionally, students will be provided with five scholarly articles pertaining to advances in prehospital treatment. Students must read "Optimizing the Patient Handoff Between EMS and the ED" and may pick two of the remaining four articles. Worksheets must be completed for the three articles.<br><br>On the final morning of the elective, students will give a 15–20-minute case presentation about an interesting or unique call that they went on (see presentation checklist).
Grading	Pass/Fail<br><br>By the end of the two weeks, students will be expected to have completed and submitted:<br>☐ Student Skill Tracking Form<br>☐ Patient Encounter Log<br>☐ Three scholarly article worksheets (one required, two of the student's choice)<br>☐ Open Book Protocol Test<br>☐ Case Presentation
Recommended Texts	Articles to be read include:<br>o "EMS Makes a Difference: Improved Clinical Outcomes and Downstream Healthcare Savings. A Position Statement of the National EMS Advisory Council." Annals of Emergency Medicine 57.2 (2011).<br>o Galvagno, Samuel M., Elliott R. Haut, S. Nabeel Zafar, Michael G. Millin, David T. Efron, George J. Koenig, Susan P. Baker, Stephen M. Bowman, Peter J. Pronovost, and Adil H. Haider. "Association Between Helicopter vs Ground Emergency Medical Services and Survival for Adults With Major Trauma." JAMA 307.15 (2012): 1602-1610.<br>o Greene, Jan. "EMS and Information Sharing." Annals of Emergency Medicine 64.2 (2014): 15A-17A.

	○ Kahn, Christopher. "Commentary: If We Shoot Ourselves in the Foot, Will EMS Be There to Respond?" Annals of Emergency Medicine 60.6 (2012): 800-802.<br>○ Meisel, Zachary F., Judy A. Shea, Nicholas J. Peacock, Edward T. Dickinson, Breah Paciotti, Roma Bhatia, Egor Buharin, and Carolyn C. Cannuscio. "Optimizing the Patient Handoff Between Emergency Medical Services and the Emergency Department." Annals of Emergency Medicine 65.3 (2015): 310-317.<br><br>• Additional resources:<br>○ FEMA Handbook for EMS Medical Directors https://www.usfa.fema.gov/downloads/pdf/publications/handbook_for_ems_medical_directors.pdf<br><br>○ National Association of EMS Physician Position Statements https://naemsp.org/resources/position-statements/
Additional Information	Dress Code: When acting as a ride along, students may wear either khaki or cargo pants with a dark colored collared shirt, or dark colored EMS BDU pants with a collared shirt or blank button up uniform shirt. Closed toe shoes are required.<br><br>BDU example: http://www.galls.com/propper-genuine-gear-bdu-trousers?PMWTNO=000000000002065&PMSRCH=#.VaVqBpNViko<br><br>Uniform shirt example: http://www.galls.com/lawpro-100-polyester-short-sleeve-shirt?PMWTNO=000000000002180&PMSRCH=#.VaVqQJNViko

Appendix B: Pre-Elective Survey

Prehospital Emergency Medicine Elective Pre-Course Survey

Please complete the following survey to the best of your ability. Your answers are anonymous.

1. In addition to my work as a medical student, I was/am a: (check all that apply)
   a) EMT-Basic
   b) EMT-Critical Care
   c) Paramedic
   d) Lifeguard
   e) Other: ________________________

2. My current field of choice for residency is: ________________________________

Please circle the most correct answer for each of the following questions:

1. In NY, the highest level of 911 prehospital care is provided by:
   a) EMT-Basic
   b) EMT-Paramedic
   c) EMT-Critical Care
   d) EMT-Intermediate

2. In NY, EMT-Basics are allowed to administer all of the following medications except:
   a) Epinephrine
   b) Nitroglycerine
   c) Morphine
   d) Albuterol

3. In NY, EMT-Basics are allowed to perform all of the following skills except:
   a) Spinal motion restriction
   b) IV insertion
   c) Blood glucometry
   d) Semi-automated Defibrillation

4. In Suffolk County, Paramedics are allowed to administer all of the following medications except:
   a) Cardizem
   b) Succinylcholine
   c) Versed
   d) Propofol

5. In Suffolk County, Paramedics are allowed to perform all of the following skills except:
   a) Pericardiocentesis
   b) Needle thoracostomy
   c) Needle cricothyroidotomy
   d) Synchronized cardioversion

6. Prehospital care providers work under the medical license of:
    a) their supervisor
    b) their medical director
    c) the patient’s primary care physician
    d) the physician receiving the patient at the hospital

7. In New York, a paramedic’s initial education is at least how many hours:
    a) 150
    b) 300
    c) 500
    d) 1000

8. In New York, an EMT-Basic’s initial education is at least how many hours:
    a) 150
    b) 300
    c) 500
    d) 1000

9. When a paramedic provides an intervention to a patient without calling a physician, they are using:
    a) Online medical control
    b) Scope of practice
    c) Standing orders
    d) Medical objectives

10. When a paramedic is on scene and wants to perform an intervention that is not specifically mentioned in their protocols, they must:
    a) Call their medical director
    b) Call medical control
    c) Call their supervisor
    d) Call their patient’s primary care physician

Appendix C: Introduction E-mail to EMS Preceptors

Hi all,

We have two medical students who will be joining us for ride-alongs the next 2 weeks.

I have attached the skill sheet that gives them some reminders about things to do during their time with you all. In addition, they have plenty of things assigned to keep them entertained during down time- an open book protocol exam to get them to at least see the protocols, as well as several articles they need to read. These are senior medical students who have completed their required 2-week long Emergency Medicine clerkship, and have voluntarily chosen to come spend time with EMS, so generally the interest/engagement level is pretty high. By the end of the two weeks, often times they describe the experience as "eye opening", which I think is a great testament to you all!!

They will have an N95 and eye protection with them, but please ensure sure they're continuously PPE-safe. If there are any issues, please don't hesitate to reach out to me in real time (XXX-XXX-XXXX). Some good skills for them to help out with are supplementary oxygen administration, learning how to do a 12-lead/getting the patient on the monitor, blood glucose, and can start an IV. They should not be placing IOs or intubating patients. Interestingly enough, drug math is not emphasized as much in medical school as it is in paramedic school, so reviewing common drug math may also be something they would be super appreciative to learn about.

THANK YOU for the extra energy you put into chatting with them and sharing your experiences- you all go above and beyond, and it's very much appreciated!

[Attach E. Student Skill Tracking Form.docx]

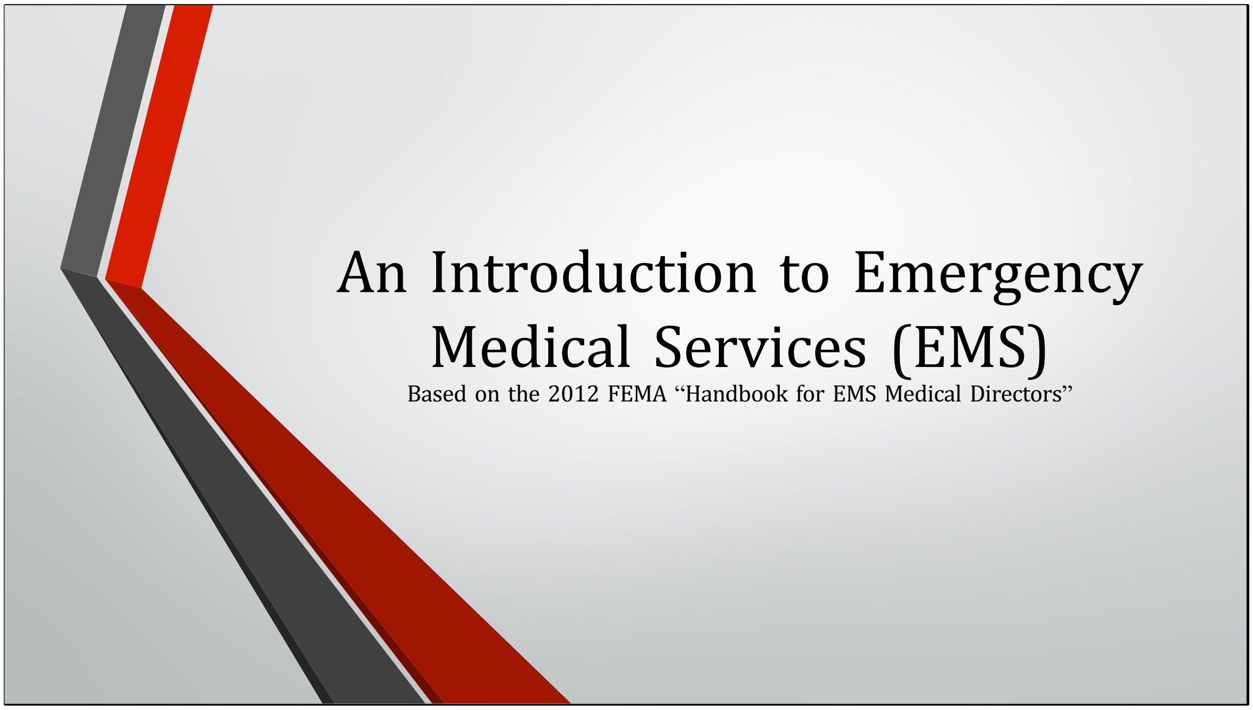
An Introduction to Emergency Medical Services (EMS)
Based on the 2012 FEMA "Handbook for EMS Medical Directors"

Goal

- Raise awareness among future physicians about the impact that prehospital care, rendered by Emergency Medical Services (EMS), can have on their patients' outcomes.

And have fun.

Objectives

- By the end of the presentation, the medical student will be able to:
  - Summarize the evolution of modern EMS
  - Describe the basic organizational structure of EMS
  - Name three advanced interventions a paramedic can perform
  - Explain two ways a physician can interact with an EMS provider to foster professional development and mutual respect

Evolution of EMS

- Mid 1900s: Funeral home hearses used to transport patients to the hospital; no care rendered, hope for the best…
- WWII: Development of the combat medic -> model for civilian paramedic
- Korean and Vietnam Wars: use of helicopters to rapidly evacuate casualties -> civilian medevac programs
- 1966: “Accidental Death and Disability: The Neglected Disease of Modern Society” -> trauma/traffic deaths due to poor emergency medical care
- 1966: “National Traffic and Motor Vehicle Safety Act” ->
  - Develop standards for treatment of MVA trauma patients
  - Creation of National Highway Traffic Safety Administration (NHTSA)
    - Create/implement EMS legislation, education standards, funding

Combat medic model- limited medical evaluation and treatment brough to site of injury.
Civilian paramedic role made for combat medic returning home from war.
Then started to evacuate patients from one location and then stabilize before transporting to hospital.
Legislation developed to start taking care of MVA patients.
Up until recently, only way to bill was based on mileage.

Evolution of EMS

- 1970: National Registry of Emergency Medical Technicians (NREMT) was founded -> national certification agency
- Early 1970s: TV show Emergency! raises public awareness about EMS
- 1973: “EMS System Act of 1973” -> define an EMS system
  - “[An EMS system] provides for the arrangement of personnel, facilities, and equipment for the effective and coordinated delivery of health care services in an appropriate geographical area under emergency conditions… and which is administered by a public or nonprofit private entity...”[3]
  - 14 critical components: integration into the health-care system, EMS research, legislation and regulation, system finances, human resources, medical direction, education systems, public education, prevention, public access, communication systems, clinical care, information systems, evaluation
- 1979: American Medical Association (AMA) officially recognizes:
  - Emergency Medicine as a Medical Doctor Board Specialty
  - Emergency Medical Technician and Paramedic as allied health occupations

Eversole, J/M. 2003. The Fire Chief’s Handbook (6th ed.) Tulsa: PennWell Corp.

1970- Started to develop a way to certify people.
EMS Act of 1973- more specific framework for EMS infrastructure.

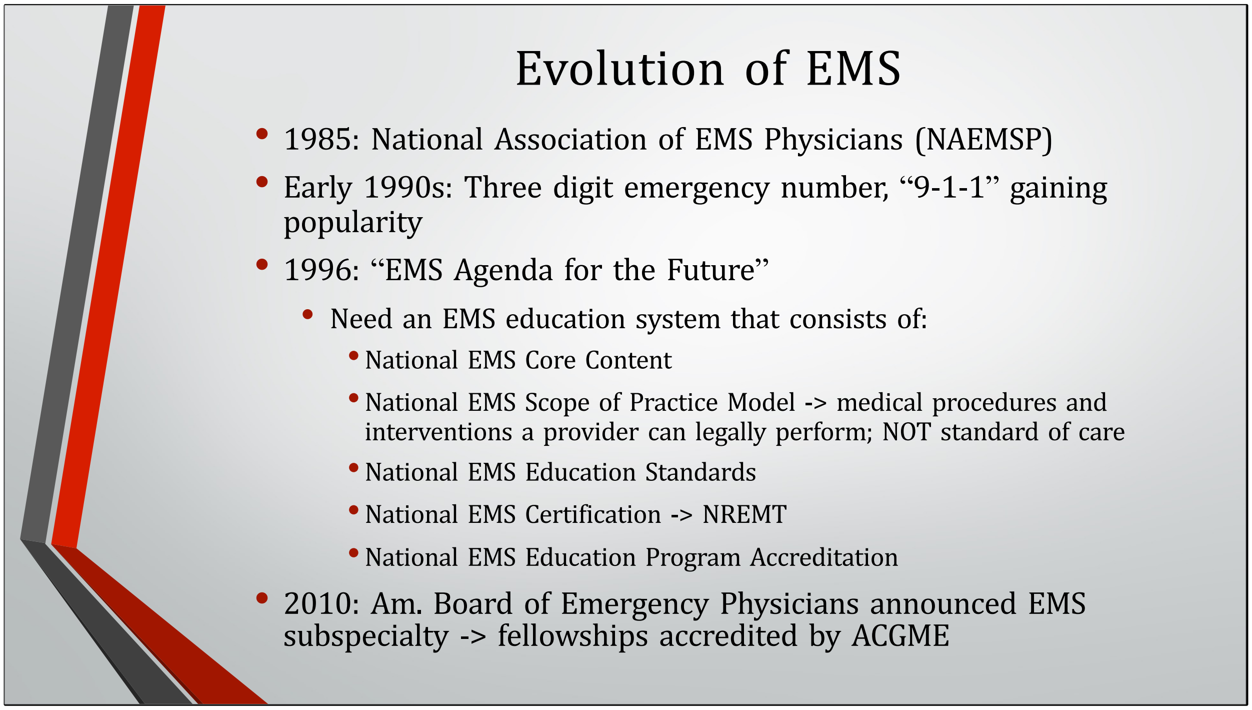

Eversole, J/M. 2003. The Fire Chief’s Handbook (6th ed.) Tulsa: PennWell Corp.

Standard of care = “established by identifying the level of care provided by equally trained personnel given the same situation.”

SOS message from cell phones- can give x, y and z coordinates
Initially, each state could make their own rules and regulations.
1996- National standards created for EMS education system

EMS Agenda 2050

- Following community input, regional meetings around the country and discussion, the EMS Agenda 2050 was released in 2019
- Changed the patient-centered focus to include not only the patient, but the patient's family, community and the EMS clinicians
- While difficult to predict the clinical and technological capabilities that will be available in 2050, the goals of EMS systems at that time will be based on six guiding principles

https://www.ems.gov/issues/planning-for-the-future-ems-agenda-2050/

EMS Agenda 2050

- Goals of EMS systems in 2050 will be based on these 6 guiding principles:
  - Adaptable and innovative
    - Test ideas in a safe and systematic way and implement effective new programs
  - Inherently safe and effective
    - Minimize exposure of people to injury, infections, illness or stress
  - Integrated and seamless
    - Local EMS services collaborate frequently with community partners and communication and coordination across the care continuum are seamless
  - Reliable and prepared
    - EMS systems are prepared for anything by being scalable and able to respond to fluctuations in day-to-day demand as well as major events
  - Socially equitable
    - Access to care, quality of care and outcomes are not determined by age, socioeconomic status, gender, ethnicity, geography or other social determinants
  - Sustainable and efficient
    - EMS systems have the resources they require to provide care in a responsible and sustainable framework that appropriately compensates clinicians

https://www.ems.gov/issues/planning-for-the-future-ems-agenda-2050/

Modern EMS

- "The practice of providing emergency care that is remote from a health-care facility, in all of its complexities[1]"
  - 911: entry into the health-care system during an emergency
    - Public dials 911 -> EMD triages/codes-> appropriate response based on local structure
  - Interfacility: patient "movement between healthcare facilities[2]"

1: National Highway Traffic Safety Administration (NHTSA). 1996. "EMS Agenda for the Future". Washington, DC: Department of Transportation (DOT)
2: Handbook for EMS Medical Directors, FEMA, March 2012.

Along with 911 response, modern EMS has many other roles including interfacility transport.
Different skill set and equipment needed for interfacility care which is not part of initial paramedic education.
Mobile Stroke Unit- bringing CT machine and medications to the patient's bedside.
Community Paramedicine- how can we help reduce patient's needing to come to the hospital
Example- how can we do well checks at home for CHF patients- reviewing medications and checking weight to help keep them from coming to the hospital for exacerbations. Reduces health care costs and improves quality of life.

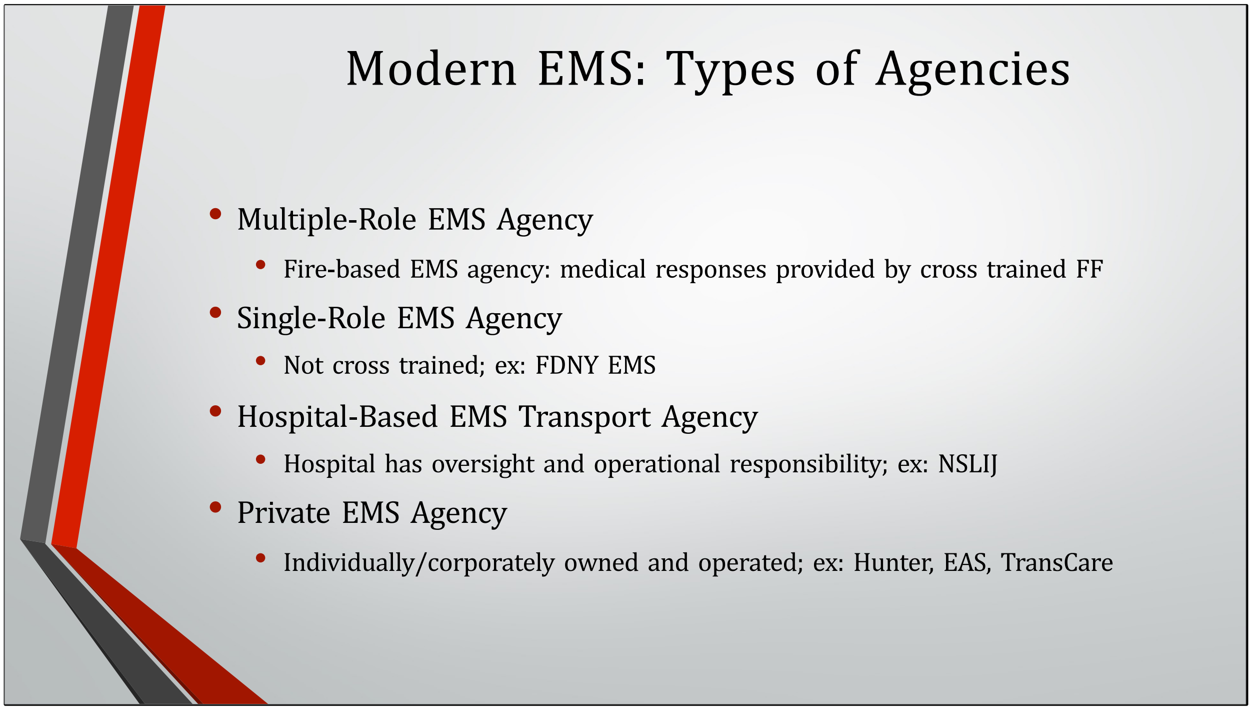

FF = fire fighters

Multiple-Role EMS agency- everyone is cross-trained to do everything. Expensive to train everybody and maintain credentials.
Single-Role EMS agency- trained for specific role. A lot of larger city programs.
Private EMS agency- paid minimum wage, do a lot of discharges from the hospital and taking patient to dialysis appointments. Help to transport patients out of the hospital to reduce bed boarding.

Modern EMS: Staffing Types

- Basic Life Support (BLS)
  - Emergency Medical Technician-Basic (EMT-B)
- Advanced Life Support (ALS)
  - Paramedic (EMT-P)
  - *in NY: Emergency Medical Technician- Critical Care (EMT-CC)

- Paid/Career vs Volunteer
- Single responder (fly car) vs two providers in ambulance (BB, PB, PP)

BB: double EMT-B
PB: EMT and Paramedic
PP: double paramedic

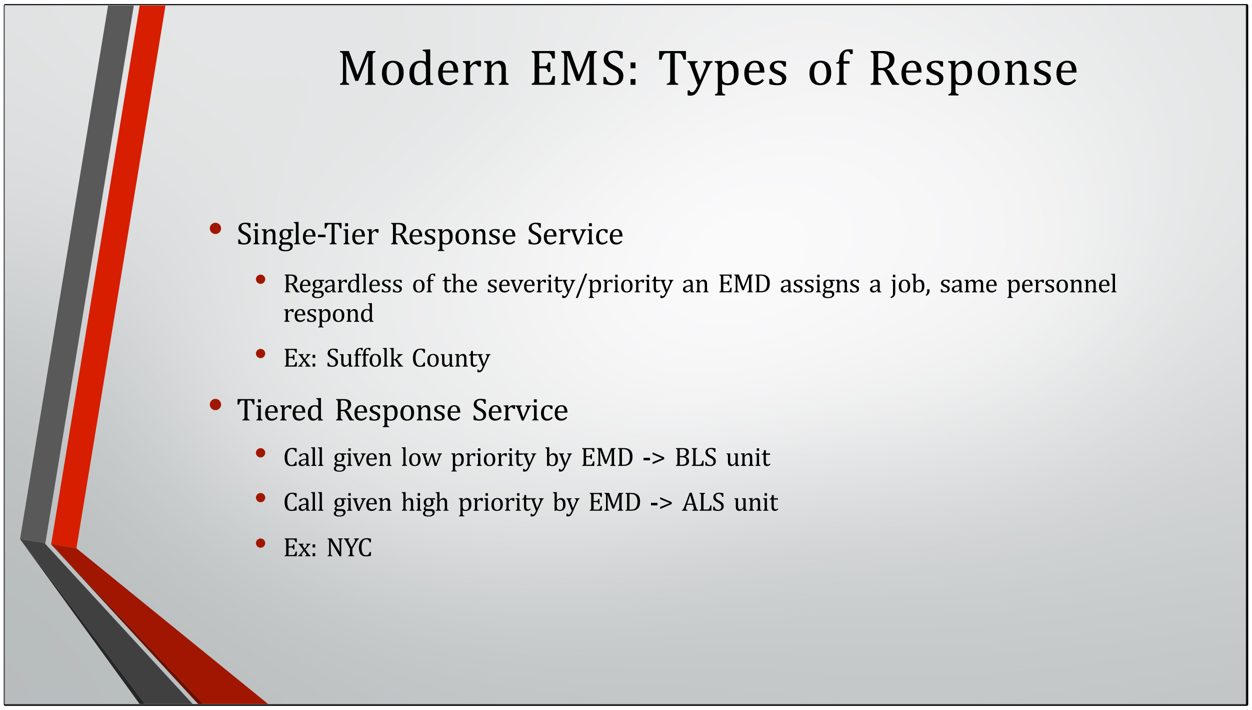

EMD = emergency medical dispatch

Dispatcher reading from set of cards to help triage/code the call- assigning call type/response.
Single-tier Response Service- same resources dispatched for every call regardless of severity.
Tiered Response Service- Different level of resources dispatched based on severity. BLS vs ALS unit.

Modern EMS: Additional Considerations

- Ground vs air transport (911 and interfacility)
- Disaster Management
- Mass Casualty Incidents
- Technical Rescue
- Tactical EMS
- Wilderness EMS

- Role of the Medical Director
  - Offline medical direction: written protocols that describe “standing orders”- i.e. what a provider can do on their own without having to call
  - Online medical direction: when a provider speaks to a physician to get verbal orders or receive advice/direction on how to manage an unusual or complex patient
  - Field Physician

Overview of Provider Education

- 1. Initial Class (EMT-B, EMT-CC, EMT-P)
- 2. Pass State/National Practical and Written Exams -> certification
- 3. If ALS -> pass regional credentialing exam (NYC, Suffolk, Nassau)
  - Essentially prove that you know the local protocols
- 4. Gain employment in agency -> function under Medical Director's license

- Every 2-3 years, must recertify/refresh by continuing medical education hours or by retaking certification exam

Not every paramedic is allowed to perform RSI. Have to go through extra training and become credentialed and then function under Medical Director's license.

EMS “Hierarchy”

- Medical Director, EM Physician
- *Certified Flight Paramedic (FP-C)
- *Critical Care EMT-P (CCEMT-P)
- **Paramedic**
- **EMT-CC
- ***EMT-Intermediate/**Advanced EMT** (**AEMT**)
- **Emergency Medical Technician (EMT)** (formerly EMT-Basic)
- **Emergency Medical Responder (EMR)**

- **Nationally Recognized Provider Levels**
- *Additional certifications for experienced paramedics; beyond entry level
- **Only recognized in certain regions of NY
- *** Recognized in some states, excluding NY

EMR- basic first aid training- firefighter, police office, security guard
Paramedic- highest level of care that is not a physician in 911 setting
Interfacility settings- additional education for critical care paramedic or certified flight paramedic
Medical Director is not always EM physician. For example, if it is a neonatal interfacility transport program, there is going to be NICU attending as the medical director.

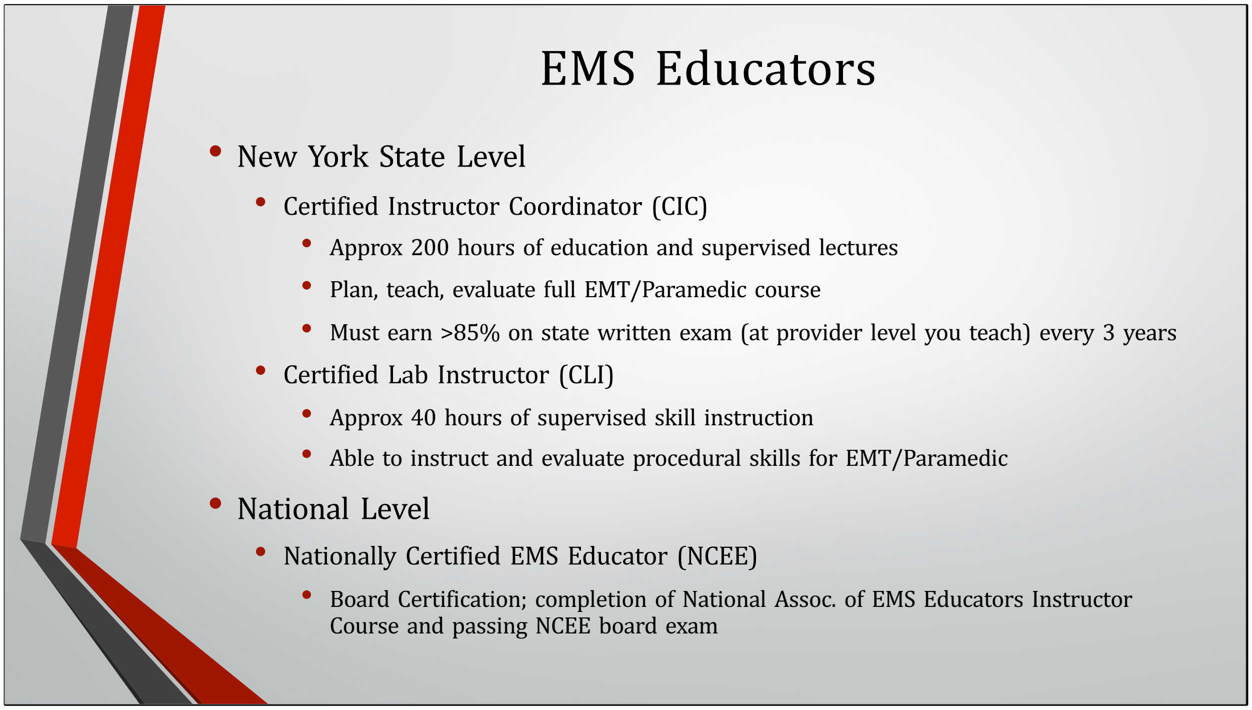

In order to be an instructor, you need hundred of hours of supervised training, teaching and verification of skills.
In NYS, there are 2 levels- CIC and CLI.
At a national level, there is an equivalent- NCEE. Also involved in writing curriculums and validating test questions.

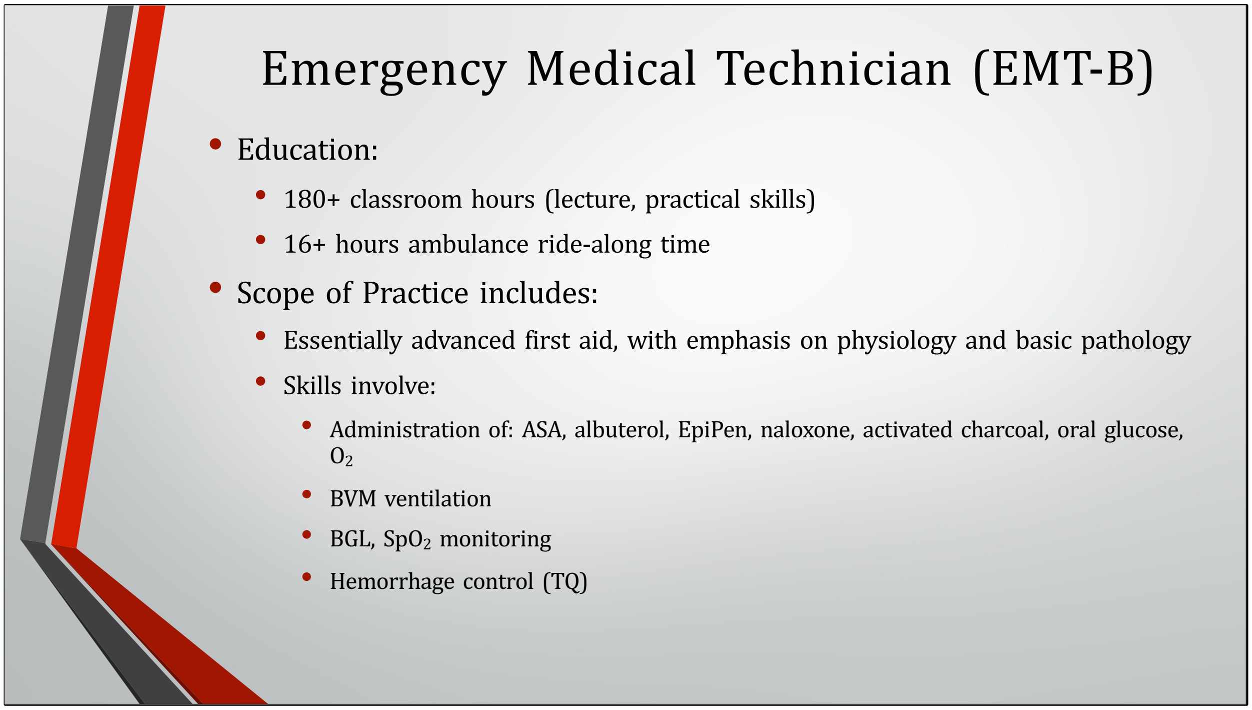

Only 16 hours of ambulance ride-along time required.

Scope of practice is essentially advanced first aid: administration of asa, albuterol, epipen, Narcan, blood glucose, oxygen, pulse oximetry and tourniquet application.

Paramedic (EMT-P)

- Education:
  - 700+ classroom hours (lecture, practical skills)
  - 700+ clinical hours (ambulance, ER, OR, MICU, SICU, PICU, NICU, CACU, L&D)
- Scope of Practice includes:
  - Skills:
    - IV/IO
    - Manual defibrillation
    - Synchronized Cardioversion
    - Transcutaneous pacing
    - Endotracheal intubation
    - CPAP
    - 12 lead EKG interpretation
    - Needle decompression
    - Needle cricothyrotomy
    - *RSI
    - *transport ventilator
    - *IV infusion pump
  - Medications:
    - Fentanyl, morphine
    - Ativan, valium, versed
    - Epi, dopamine
    - Amiodarone, metoprolol, cardizem
    - Solumedrol, solucortef
    - Magnesium sulfate
    - Albuterol, ipratropium bromide
    - Diphenhydramine
    - D50, CaCl, HCO3
    - Adenosine, atropine
    - Naloxone
    - Lasix
    - Glucagon, thiamine
    - Etomidate
    - Zofran
    - NTG, ASA
    - Haldol
    - Toradol

At least 700 hours of clinical hours in ambulance, ER, all the ICUs, L&D, sometimes cath lab.
Generally 10-12 month program.

Benzos, narcotic, antidysrrhythmics, cardiac arrest medications, paralytics.

System Example: NYC EMS

- Ambulances are staffed with 2 people
- Ambulances are "posted" in strategic locations to meet call volume
- Crew waiting to respond to 911 call

- Serious calls: ambulance with 2 paramedics respond
- Not so serious calls: ambulance with 2 EMT-Bs respond
- Keep in mind, "serious" v "not so serious" is determined by the information provided to the police dispatcher, who then "turfs" the call to the EMS dispatcher once it has been determined it is an EMS call

Different cities will have different models.
Strategic locations in different parts of the city waiting for calls.
Can shift around the units that are in service so that they are not coming from a huge distance.

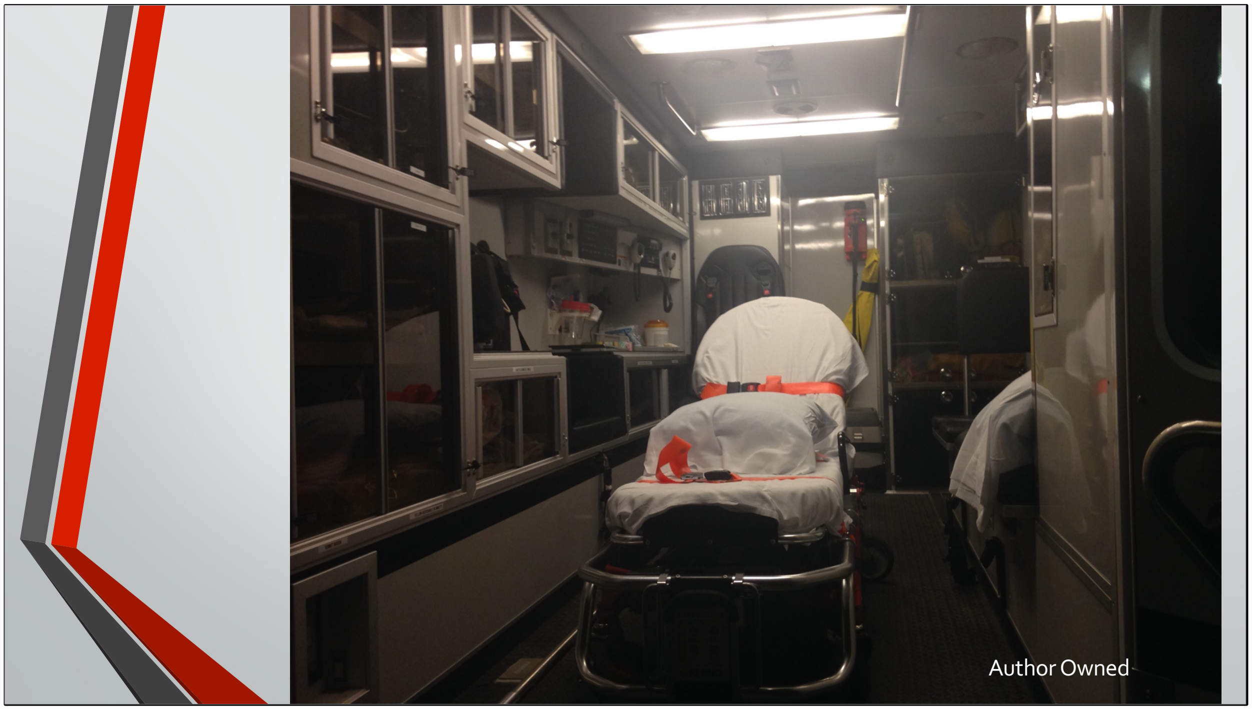

Talk about what supplies are where, ex airway supplies are located in the cabinets near the head of the stretcher

System Example: County EMS

- Originally, EMS provided by volunteers, who were notified via pager of a call, and then responded to the FH to get the ambulance and then go to the call
- Now, most towns have paid first responders on duty (close to) 24/7. We are at the FH and respond to calls directly via an SUV
- Volunteers meet us on scene with an ambulance

In general, there is a provider at a fire house.
Fire district lines can be obscure.

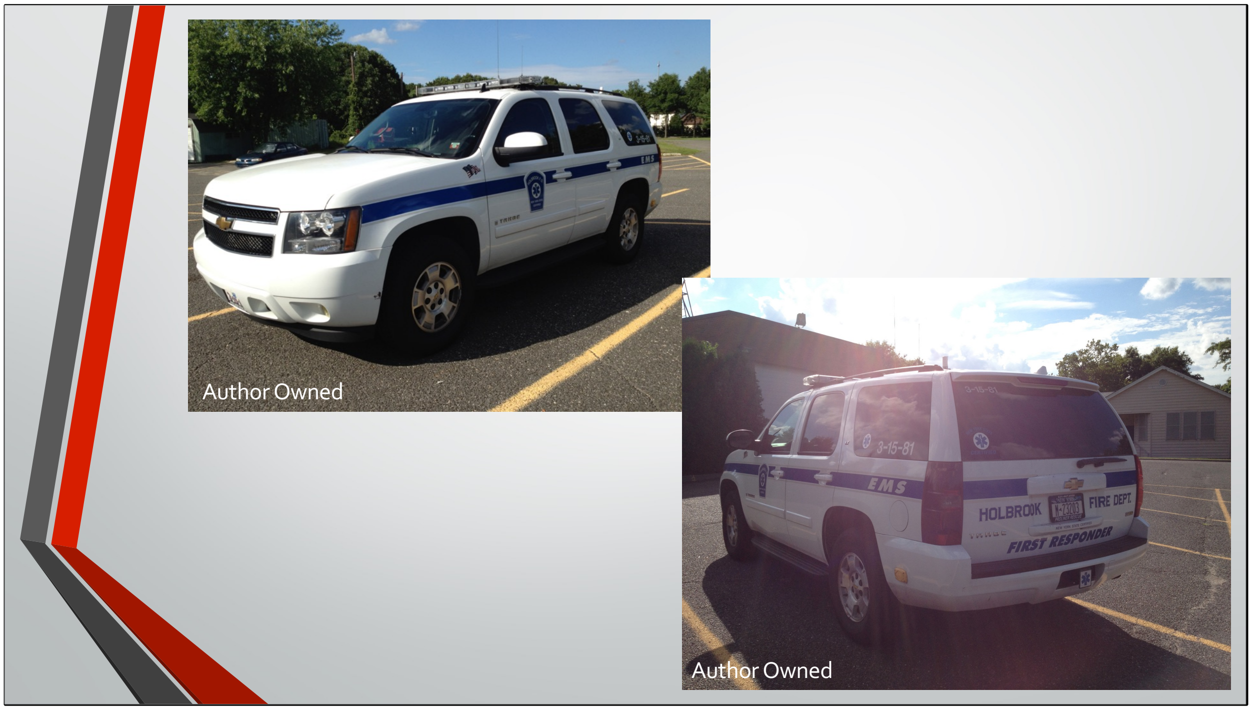

Example of fly care packed with equipment.

Paramedic (EMT-P)

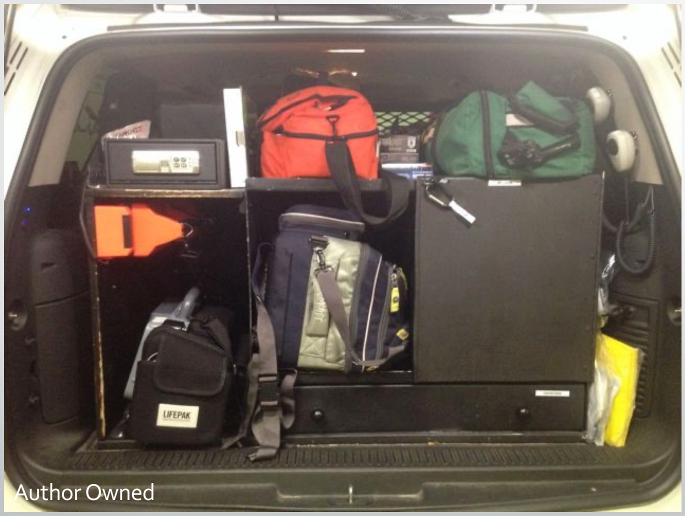
Author Owned

70-75lbs of gear.
Cardiac monitor, drug bag, oxygen bag, BLS bag, suction machine.

Paramedic (EMT-P)

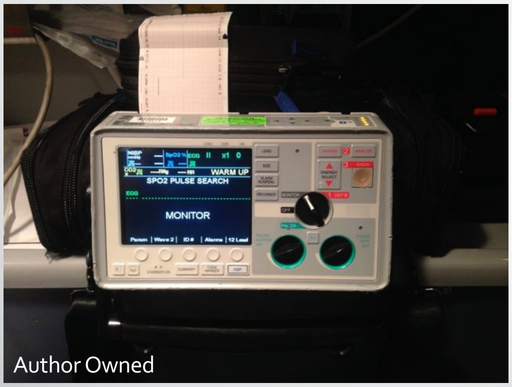

Author Owned

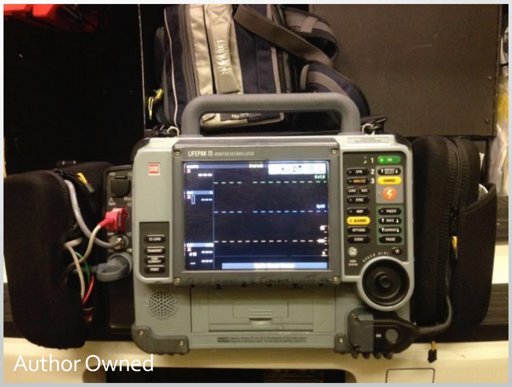
Author Owned

Different types of monitors, important to know how to use them.

Paramedic (EMT-P)

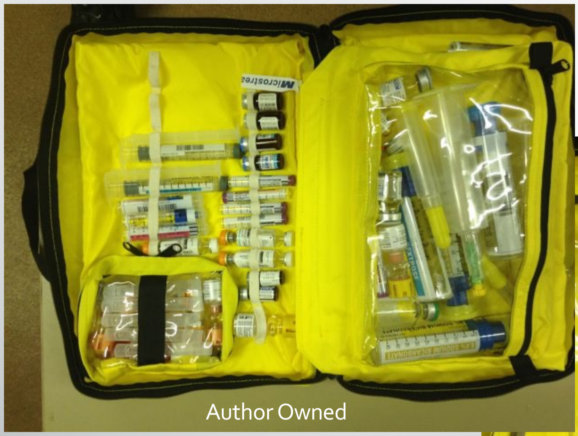

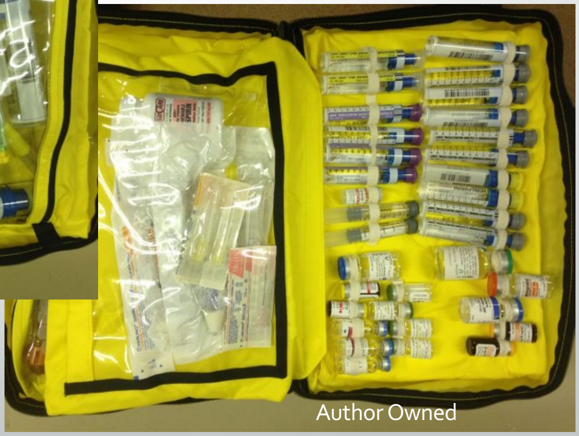

Pros and Cons.
Medications can look very similar especially in the dark. Do not have safety mechanism of Pyxis.
Need to be attentive to detail and ensure you are drawing up the right medication.

Paramedic (EMT-P)

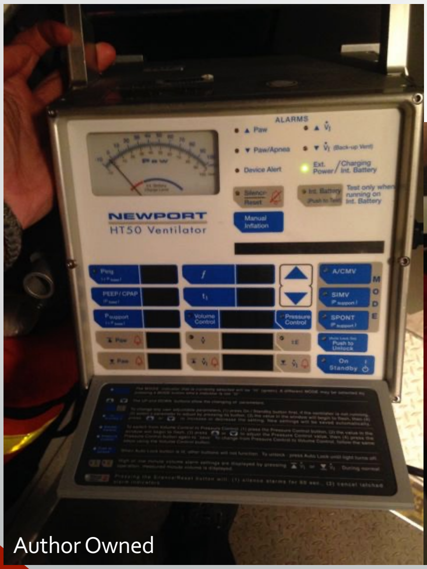

Author Owned

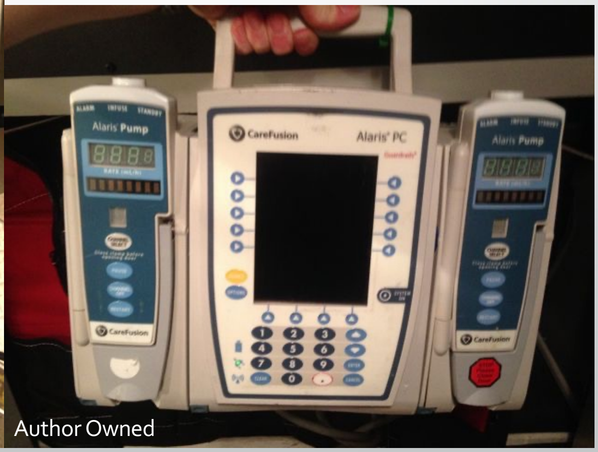

Author Owned

Different types of vents.
Infusion pumps- Ilaris is one of the most common types.
Learn how to use infusion pumps.

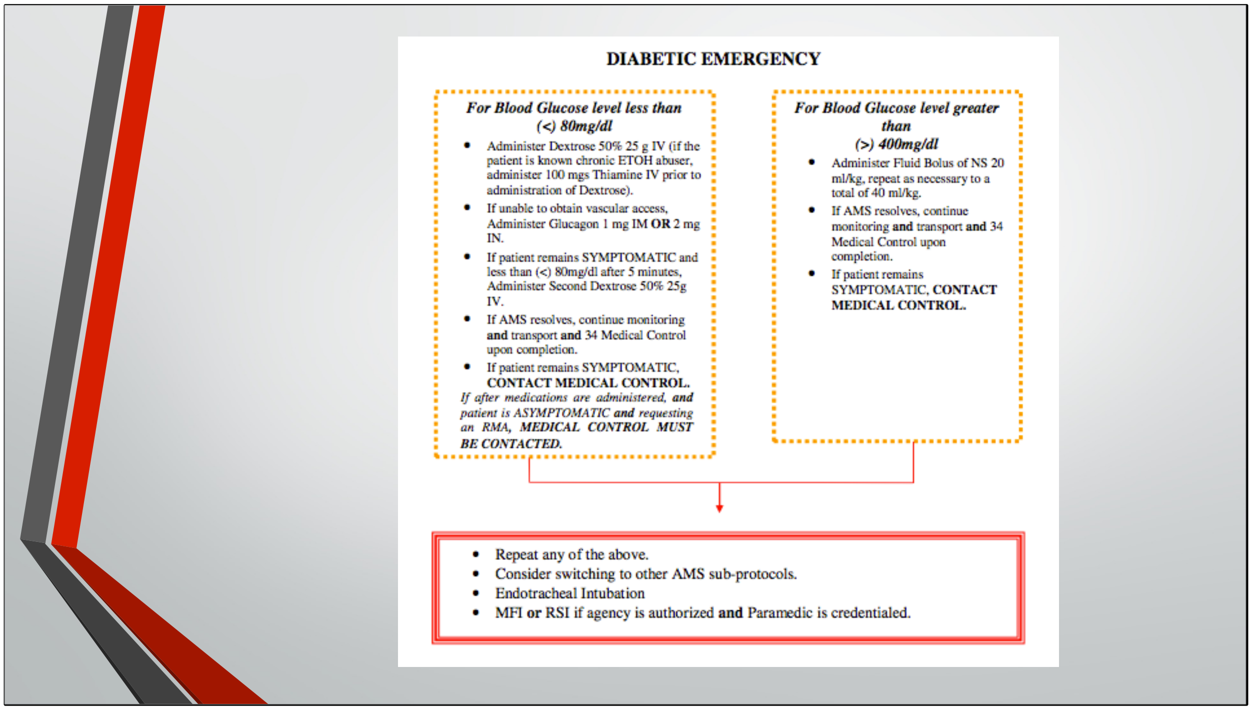

Example of a protocol.
This is how Suffolk county organizes there protocols- color coded for what type of provider can do what on their own before having to call medical control.

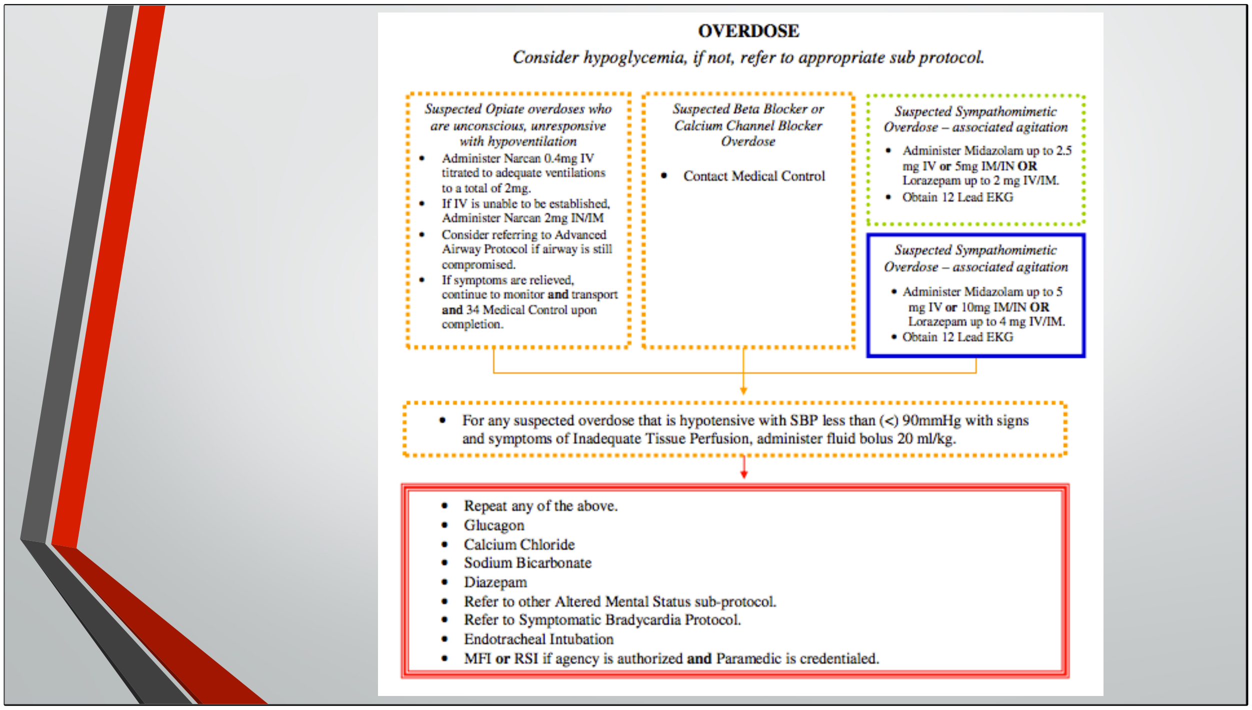

Comment on doses as compared to what might be given in the hospital

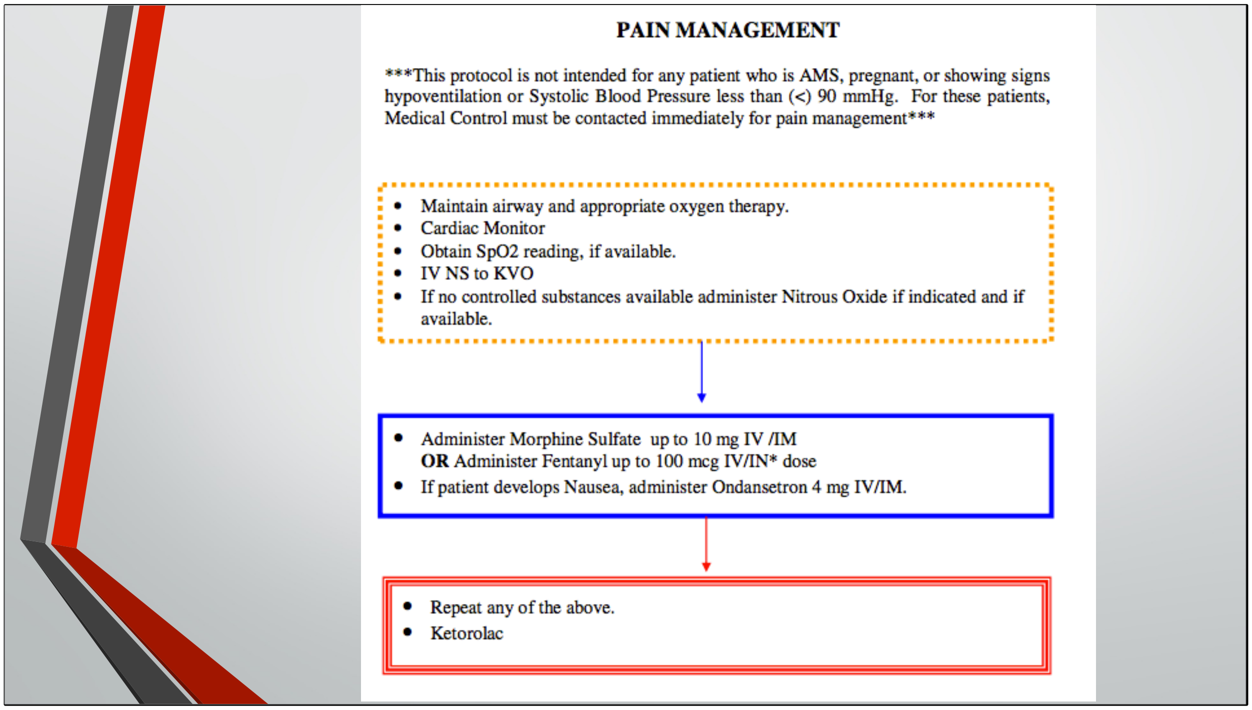

PAIN MANAGEMENT

This protocol is not intended for any patient who is AMS, pregnant, or showing signs hypoventilation or Systolic Blood Pressure less than (<) 90 mmHg. For these patients, Medical Control must be contacted immediately for pain management

- Maintain airway and appropriate oxygen therapy.
- Cardiac Monitor
- Obtain SpO2 reading, if available.
- IV NS to KVO
- If no controlled substances available administer Nitrous Oxide if indicated and if available.

- Administer Morphine Sulfate up to 10 mg IV /IM
  OR Administer Fentanyl up to 100 mcg IV/IN* dose
- If patient develops Nausea, administer Ondansetron 4 mg IV/IM.

- Repeat any of the above.
- Ketorolac

Morphine and fentanyl for pain management.

Come on a Call

- Dispatch info: “Respond to a male, cardiac at 123 Fey St. Time out: 0317”
- You have a 4 minute drive, lights and sirens, to the residence. You must navigate using GPS, control the sirens, and listen/respond to radio as you drive. Assume you are the sole paramedic first responder.
- What are you thinking on the way over?

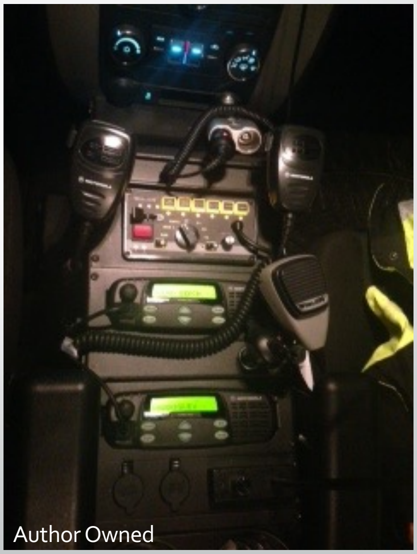
Author Owned

Model of an actual call to give you a sense of time line, work flow and expectations.
Woken out of sleep to a call at 0317 AM.
Thinking about what questions am I going to ask first, what other resources might I need, is this something I am going to need to call for back up? Do I know where I am going, do I need to plug this into GPS? Where will I be going with this patient?

Come on a Call

- After arriving on the block, you have difficulty locating the house, as the house number is not visible from the street, and it is pitch black out
- Patient’s wife sees the flashing lights, and turns the front porch light on- the only light on the block.
- You hear dogs barking...

May have difficulty finding the house numbers- wreaths may be blocking house number, mailbox may be on opposite side of the street.
Never run, it is important for us to have situational awareness and make sure that we are walking into a safe scenario. In EMS, it is our safety first.
Hear dogs barking, not going in until the dogs are secured.

Come on a Call

- This is the house... What are your thoughts?

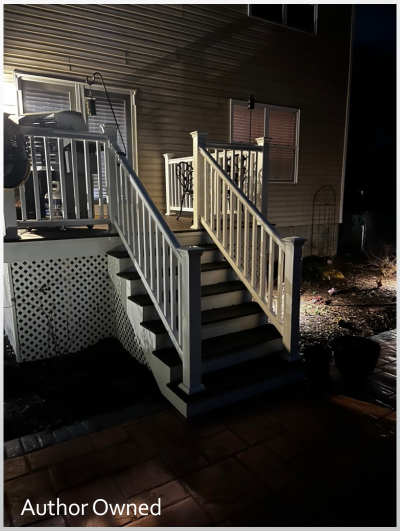

Always know your exit strategy, making sure there is nothing separating you from a door.
Technical challenges- Steps, 90 degree turns, cars in the driveway, lighting issues
How am I getting all my gear in the house and then getting the patient out?
Is there ice, gravel, rain?

Come on a Call

- You enter the house, and the patient's wife states "He's upstairs in our bedroom. He can't walk."
- Thoughts?
- Keep your exit strategy in mind… how will this change possible care you provide upstairs v in ambulance?

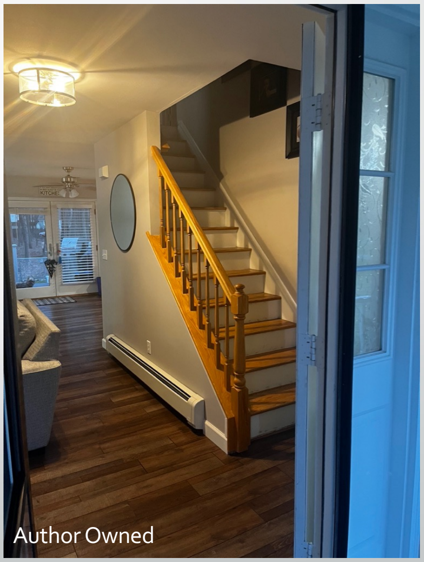

Patient may be upstairs and not able to walk. Turns, floor mats, slip risks. How am I safely going to get this patient out of the house?
If the patient needs CPAP, am I going to start that upstairs and then have to carry oxygen cylinder down? Am I starting the cardiac monitor upstairs or waiting until I get them down?
Where am I going to start care? Not something we think of in the hospital.

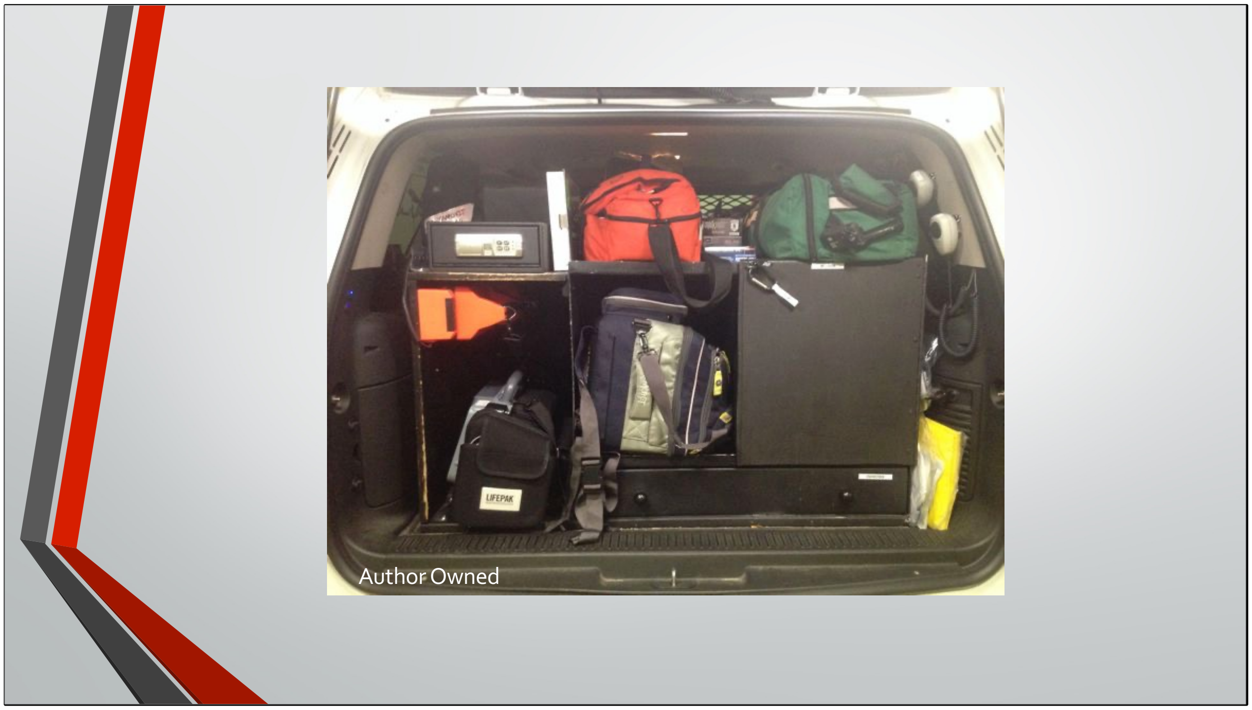

Again remembering that you have to get all of your equipment in and then you have to get the patient out.

Come on a Call

- Your patient is a 67 year old male, alert and oriented, sitting up in bed, complaining that "my chest hurts"
- DDX?
- Questions to ask?
- (PS- you have <10 minutes to do a H&P)

You finally make it to your patient.
What questions do you ask? What is different than the workflow in the hospital, is that you have about 10 minutes to multitask and get an H&P, start an IV and get the patient on the monitor.

Come on a Call

- HPI: 9/10 non-radiating retrosternal CP, no radiation, unchanged by rest or movement. Gradual onset 12 hours ago, SOB x 4 hours. Pt denies any recent illness, trauma, or family hx. Pt finally called 911 "because my wife won't stop nagging me, and now I can't sleep"
- PMH insignificant other than smoking 1 pack a day x 50 years
- No medications, allergic to PCN (-> hives)

Come on a Call

- VS: 98/60, HR 102, SpO2 92% ORA -> 96% on 2 LPM NC
- PE: Remarkable for skin that’s pale, cool, diaphoretic
- NSR on monitor

Soft blood pressure, slightly tachycardic, not satting well on room air. Remember that you may have poor lighting for physical exam.

Come on a Call

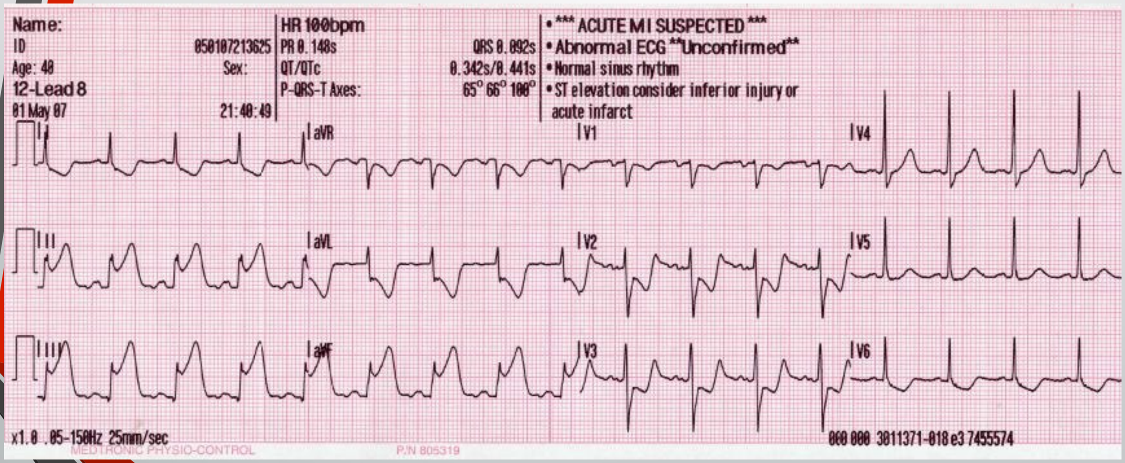

http://commons.wikimedia.org/wiki/File:12_lead_generated_inferior_MI.JPG
Freely available image

"Image by [OWNER], retrieved from: [http://commons.wikimedia.org/wiki/File:12_lead_generated_inferior_MI.JPG] on [11 March 2023]. Image is in the public domain."

Massive inferior heart attack with reciprocal changes.
Now we are on the clock. Racing to get this patient to cath lab. Already going on for 12 hours. What do we need to do to get patient out of the house and to the correct hospital?

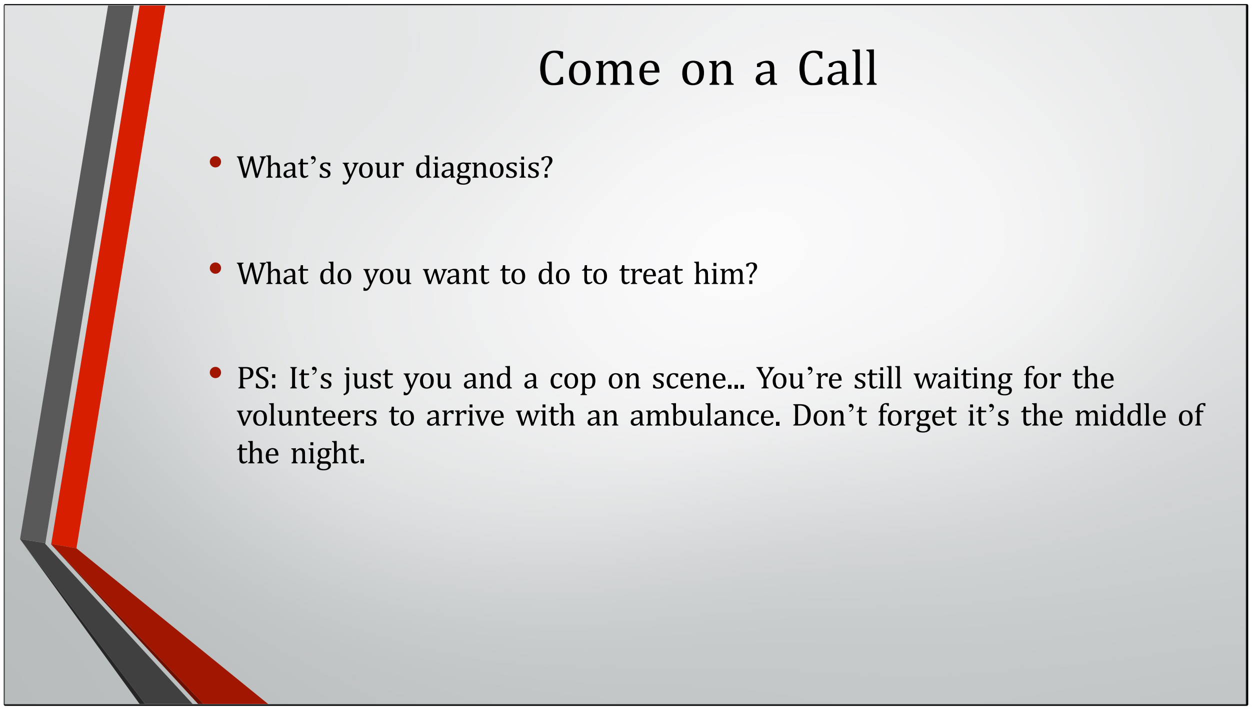

Inferior STEMI.
Going to give aspirin and fluids. Going to be careful with pain control because of soft blood pressure. Going to avoid nitro because of the STEMI pattern.

Come on a Call

- Other considerations:
  - Need to package the patient, and get him to the ambulance… After going down a flight of stairs. Are you keeping monitors and O2 attached?
  - Time management…. "Stay and play" versus "scoop and run" interventions
  - Which hospital do you go to? Bypass nearest to go to most appropriate (ie one with a cath lab)?
  - Can the family be left to fend for themselves?
  - Crowd control… As if you don't have enough to do
  - Don't forget to lock up the house!

Other considerations that we almost never think about in hospital:
Need to get him down the stairs, there is a real chance that he may go into cardiac arrest. Who is going to carry the monitor down the stairs.
"Stay and play"- if patient is in SVT- we can fix what is going on while at the scene.
"Scoop and run"- severe trauma- patient needs the OR- not much we can do on scene that is going to be meaningful.
Not every hospital can do an emergent cardiac cath.
Other things- what if the patients spouse is completely dependent on patient for care? What if there are small children?
Have to remember to get keys and lock the door.

Come on a Call

- Other considerations:
  - Can he lie flat in a Reeves to go down the stairs, or does he need to sit up in a chair?
  - What would you do if he was 350lb?

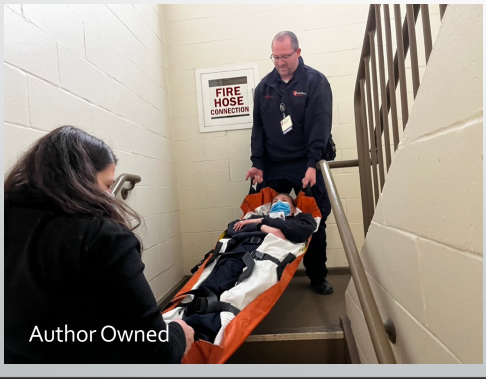

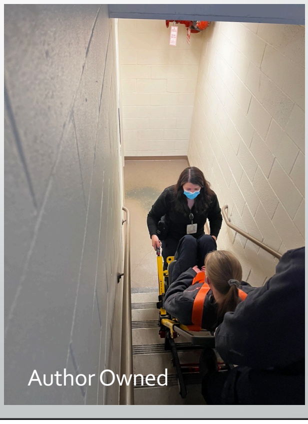

Stair chair if the patient is conscious and following commands.
If someone can not sit upright or is not following directions they get put in a Reeves which is basically a tarp with a couple planks of wood in it.
Weight limits and patient restrictions. Example- elderly patients who are extremely kyphotic and might not be able to lie flat or comfortably sit in stair chair.

Come on a Call

- Dx: Inferior wall STEMI

- Interventions: pulse oximetry, supplemental oxygen, ASA, monitor, 12 lead, IV lock x2, IVF: NS at KVO for now
- Not a candidate for NTG, or morphine due to SBP and type of MI
- Consider fluids and pressors; prepare for cardiogenic shock, cardiac arrest

- Bypassed Mather and St Charles to go to SBUH
- Code H called en-route
- Upon arrival to ER, pt transferred to hospital stretcher -> cath lab

At the time, neither Mather or St Charles were cath lab centers.
Especially on nights and weekends, a prehospital notification of 15 minutes helps to get people to the hospital.
Code H was called prehospitally. Patient went right to the cath lab.

Come on a Call

- Time Dispatched: 3:17
- On Scene: 3:21
- Patient Contact: 3:25
- 12 lead transmitted: 3:34
- Depart Scene: 3:45
- At SBUMC: 3:55
- In Cath lab: 4:04

- (In reality, this was a 3 person full paid crew; 1 medic, 2 EMT-CC)

These are the actual times for this case.
Less than 1 hour from time dispatched to time in cath lab.

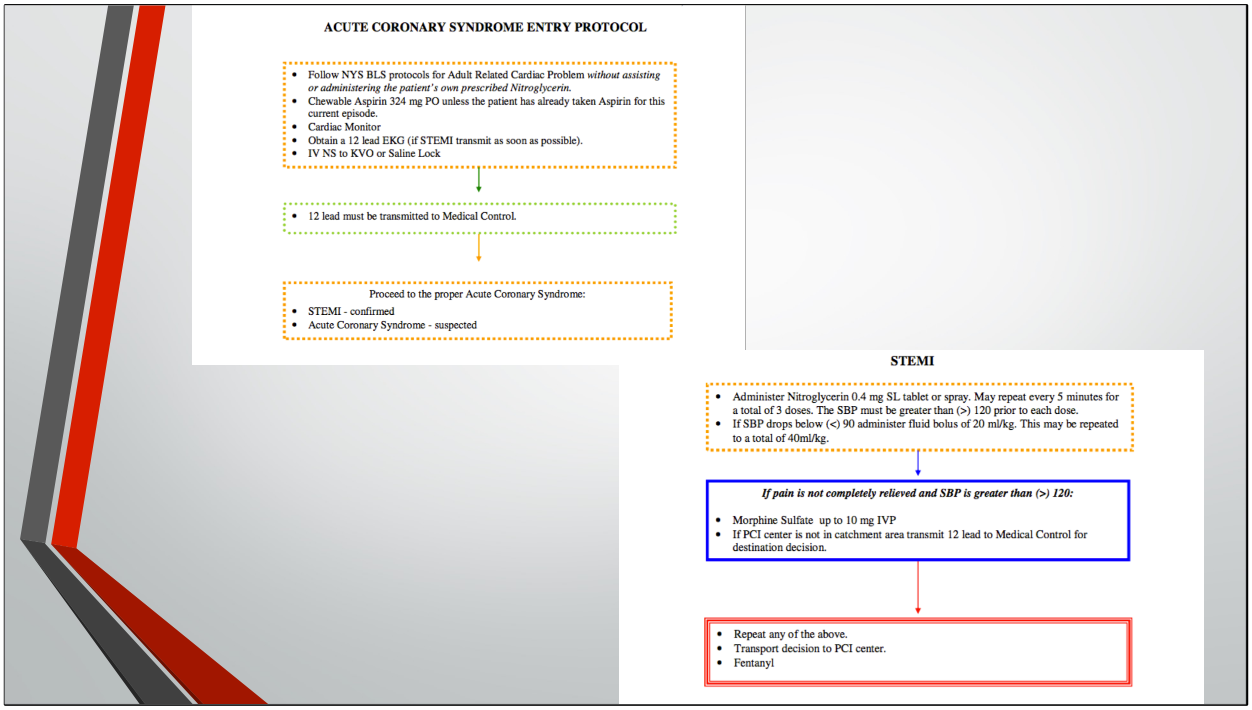

ACUTE CORONARY SYNDROME ENTRY PROTOCOL

- Follow NYS BLS protocols for Adult Related Cardiac Problem *without assisting or administering the patient's own prescribed Nitroglycerin.*
- Chewable Aspirin 324 mg PO unless the patient has already taken Aspirin for this current episode.
- Cardiac Monitor
- Obtain a 12 lead EKG (if STEMI transmit as soon as possible).
- IV NS to KVO or Saline Lock

- 12 lead must be transmitted to Medical Control.

Proceed to the proper Acute Coronary Syndrome:

- STEMI - confirmed
- Acute Coronary Syndrome - suspected

STEMI

- Administer Nitroglycerin 0.4 mg SL tablet or spray. May repeat every 5 minutes for a total of 3 doses. The SBP must be greater than (>) 120 prior to each dose.
- If SBP drops below (<) 90 administer fluid bolus of 20 ml/kg. This may be repeated to a total of 40ml/kg.

If pain is not completely relieved and SBP is greater than (>) 120:

- Morphine Sulfate up to 10 mg IVP
- If PCI center is not in catchment area transmit 12 lead to Medical Control for destination decision.

- Repeat any of the above.
- Transport decision to PCI center.
- Fentanyl

Stony Brook Medicine

Stony Brook University Hospital
Heart Institute
Department of Medicine
Division of Cardiology
Stony Brook, NY 11794-7588
[redacted] 2013
FAX: 631.444.1758

DEC 17 [illegible]

RE: Code H Feedback

Dear EMS Professionals:

Today at 3:34 am your team transmitted a 12 lead EKG to medical control which showed a STEMI and a Code H was Called. The patient was a 67 year old male with chief complaints of chest pain which began 12 hours prior. His EKG showed an inferior wall STEMI. The patient has a history of smoking.

In the cath lab the patient received an IABP for persistent chest pain and hypotension. He was placed on nitroglycerin and dopamine. His heart rate dropped to 38 and atropine was given and a temporary pacemaker was placed. In the lab the RCA showed 100% occlusion and a stent was placed reducing the stenosis to 0. The patient remained AAAx3 and is recovering in CCU.

Thank you for the great care you gave. Transmitting the EKG, especially during off shift, allows the on call team to arrive ASAP for the patient. The EKG was done at 3:19 and the patient was on the Cath table by 4:04.

Sincerely,

[redacted], RN Chest Pain Coordinator

Author Owned

Letter from SBUMCl

What can you do now as a medical student?

- Engage the provider to give you a report!
  - We see their home environment…
  - Good practice for listening to a report, thinking, and asking follow up questions in a fairly low-stress way
  - Builds cross-discipline trust

Learn how to listen to a patient presentation and ask follow up questions.

What can you do as a physician?

- Offer continuing education classes
- Educate your patient when to call 911 as it relates to their specific diagnoses
- In hospital DNR ≠ out-of-hospital DNR
- Engage crew- listen to the report, suggest your Dx and plan
  - Really tough to learn from our calls and improve our patient care if we have no idea what the “true” Dx is

Additional Resources

- NREMT: http://www.nremt.org
- NAEMSP: http://www.naemsp.org/Pages/default.aspx
- ABEM EMS Fellowship: https://www.abem.org/public/subspecialty-certification/emergency-medical-services/ems-overview
- Suffolk EMS: http://www.suffolkremsco.com/
- NAEMSE: www.naemse.org

Appendix E: Skill Tracking Form

Prehospital Emergency Medicine Elective
Student Skill Tracking Form

Skill	Date	Preceptor Signature
Review Standard Equipment (NYS Part 800) Checklist		
Change Portable O2 tank		
Assemble CPAP		
Assemble a nebulizer		
Assist with nasal cannula application		
Assist with non-rebreather mask application		
Discuss iGel<br>- How it differs from ETI<br>- How to safely remove		
Spike a bag of IVF		
Assist with placing EKG leads		
Assist with placing 12-lead EKG leads		
Assist with measuring and applying c-collar		
Discuss tourniquet application		
Discuss spinal motion restriction/transfer devices: KED, back board, Reeves		
Discuss traction splints		
Discuss IO placement (landmarks for humeral, tibial), and how to remove an IO needle		
Discuss EKG transmission to Medical Control		
Discuss Prehospital Code H activation process (EKG acquisition, transmission, hospital notification)		
Discuss Trauma Alert/Code T Criteria and activation		
Discuss RMA process<br>- High risk criteria<br>- RMA packet<br>- Capacity evaluation		
Observe Hospital Presentation<br>- Radio report<br>- Patient hand-off		
Read a completed PCR		
Read Suspected Child Abuse Packet		

Appendix F: Patient Encounter Log

EMS Elective Patient Encounter Log

Student Name: ________________________________

Location (Unit, MSU)	Date	Age, M/F	Chief Complaint	Comments

Prehospital Emergency Medicine Elective
Open Book Protocol Exam
Reference: NYS Dept of Health Bureau of EMS
Collaborative Advanced Life Support Adult & Pediatric Care Protocols
https://www.health.ny.gov/professionals/ems/pdf/ny_collaborative_protocols_v23.1.pdf

1. Which of the following is NOT a safe way to restrain a child when being transported in an ambulance:
    a. The child's own safety seat, if it is intact
    b. Integrated child restraints that are within the captain's chair/airway seat.
    c. A correctly sized child safety seat that is stocked on the ambulance.
    d. In the arms or lap of the child's parent with the parent properly restrained on the ambulance stretcher.

2. After appropriate on-scene evaluation and stabilization, patients should be transported to:
    a. The closest hospital, regardless of its capabilities
    b. The hospital the patient was born at
    c. The hospital affiliated with the ambulance company
    d. The closest appropriate hospital

3. When an advanced airway adjunct (endotracheal tube, supraglottic airway device) is placed, what is required to be utilized to confirm the correct location of the device?
    a. Continuous waveform end-tidal capnography
    b. Pulse oximetry
    c. Lung sounds
    d. 3 lead cardiac monitoring

4. EMS providers may honor all of the following except:
    a. MOLST form
    b. Non-hospital DNR
    c. DNR bracelet
    d. Hospital DNR

5. For patients in anaphylactic shock, paramedics may administer all of the following medications under standing order EXCEPT:
    a. Epinephrine 0.3mg IM
    b. Epinephrine infusion
    c. Diphenhydramine 50mg IV or IM
    d. Dexamethasone 10mg PO, IM, or IV

6. For agitated patients who are extremely combative and at immediate risk of causing physical harm to themselves or others, paramedics may perform all of the following interventions under standing order EXCEPT:
    a. Midazolam 5mg IM or IV; may repeat up to 10mg
    b. Ketamine 250mg IM
    c. Haloperidol 5mg IV or IM
    d. Application of soft restraints

7. For adult patients with symptomatic bradycardia, paramedics may perform all of the following interventions EXCEPT:
    a. Transcutaneous pacing
    b. Norepinephrine infusion
    c. Atropine 1mg IV
    d. Synchronized cardioversion

8. For patients experiencing a STEMI, what intervention can a paramedic perform that an EMT can not?
    a. Administer a bolus of IV fluids if the patient becomes hypotensive
    b. Administer ASA 324mg
    c. Transmit a 12-lead EKG
    d. Assist a patient to take their prescribed nitroglycerin if their systolic BP is >120mmHg

9. In adult patients with pulmonary edema and shortness of breath, EMTs can offer which of the following interventions, if trained and equipped:
    a. Continuous Positive Airway Pressure (CPAP)
    b. Furosemide 40mg IVP
    c. Albuterol 2.5mg in 3mL nebulizer
    d. IV placement

10. For pediatric patients with severe asthma or wheezing, which of the following interventions requires medical control approval for a paramedic to perform:
    a. Epinephrine 0.01mg/kg IM
    b. Dexamethasone 10mg PO, IM, or IV
    c. Albuterol and ipratropium nebulizers to a total of three doses
    d. Epinephrine infusion

11. In adult patients with hyperkalemia in cardiac arrest, what medication is not available for paramedics to administer?
    a. Sodium bicarbonate
    b. IV fluid bolus
    c. Insulin
    d. Calcium chloride

12. What intervention can a paramedic perform for a patient with a blood glucose reading greater than 400 mg/dL?
    a. Insulin 10 units
    b. Normal saline 500mg IV bolus
    c. Dextrose 10%, 25 grams
    d. Glucagon 1mg IM

13. For adult patients experiencing pain, all of the following medications can be administered by a paramedic without contacting a physician EXCEPT:
    a. Acetaminophen
    b. Fentanyl
    c. Ketorolac
    d. Ketamine

14. Which of the interventions is crucial for a paramedic to perform specifically if a patient who is seizing is believed to be pregnant?
    a. Magnesium 4g IV over 20 minutes
    b. Obtain a blood glucose
    c. Midazolam 5mg IV/IM/IN
    d. A second dose of midazolam 5mg IV/IM/IN

15. For patients with a suspected stroke, an EMT should perform all of the following interventions except:
    a. A blood glucose level
    b. A Cincinnati Stroke Scale
    c. A hospital prenotification
    d. Administer metoprolol for hypertension

16. Reasonable care of an amputated extremities includes all of the following except:
    a. Wrapping with sterile dressing moistened with sterile saline
    b. Placing directly on ice
    c. Placing in a water-tight container
    d. Reducing any additional trauma to the tissue

17. In the setting of unconditional bleeding of an extremity, the most optimal placement of a tourniquet is:
    a. 2-3 inches proximal to the wound
    b. 2-3 inches distal to the wound
    c. On the wound as to act as a pressure dressing
    d. As close to the torso as possible

18. In the setting of an adult patient with chest trauma with concern for tension pneumothorax who is not in cardiac arrest, which level provider is able to perform a needle decompression of the chest under standing orders?
    a. EMT
    b. EMT-CC
    c. Paramedic
    d. Advanced

19. When performing an RMA, Medical Control should be contacted if any "RMA High Risk Criteria" are present. These criteria include all of the following except:
    a. Age >65
    b. Pulse >140
    c. Serious chief complaint or significant mechanism of injury
    d. Fever in a newborn or infant less than 8 weeks old

20. Paramedics may obtain vascular access via all of these routes except:
    a. Brachiocephalic intravenous (IV)
    b. Intraosseous (IO)
    c. Internal jugular vein (IJ)
    d. External jugular vein (EJ)

Appendix H: Optimizing EMS-ED Handoff Worksheet

Prehospital Emergency Medicine Elective

Worksheet on "Optimizing the Patient Handoff Between Emergency Medical Services and the Emergency Department"

https://pubmed.ncbi.nlm.nih.gov/25109535/

1. Since *To Err is Human* was published, numerous regulatory agencies have identified patient handoffs as a high-risk event. Which of the following has not been specifically focused on:
    a. Within-hospital handoffs
    b. Posthospital discharge transitions
    c. EMS-to-ED handoffs

2. Barriers to preventing easy EMS-to-ED handoffs include all of the following except:
    a. Distinctive clinical duties
    b. Distinctive professional cultures
    c. Nonoverlapping work environments
    d. Lack of cross-disciplinary awareness

3. Participants identified all of the following as reasons why they prefer to give hand-off report directly to a physician, except:
    a. Circumvent perceived hospital provider hierarchy
    b. Prevent distortion or loss of information
    c. Potential valuable learning opportunity
    d. Facilitate follow-up on patient's ER course and outcome

4. The two concurrent hierarchies described by many participants are:
    a. Provider and acuity hierarchies
    b. Prehospital provider and receiving provider hierarchies
    c. Professional and acuity hierarchy
    d. Professional and patient hierarchy

5. Prehospital providers are more likely to give a more detailed EMS-to-ED handoff for which of the following patients:
    a. 75 yof complaining of chest pain x 5 weeks, associated with a mild cough
    b. 14 yom s/p MVA, restrained back seat passenger, ambulatory on scene, no airbag deployment, no LOC, minor exterior damage to large SUV he was in
    c. 21 yof s/p 30 foot fall, GCS 10, open bilat femur fx's
    d. 60 yom with AMS x 12 hours, RR 30, HR 110, warm to touch, dysphagia at baseline, crackles in right lower lobe.

Appendix I: HEMS v Ground for Trauma Worksheet

Prehospital Emergency Medicine Elective

Worksheet for "Association Between Helicopter vs Ground Emergency Medical Services and Survival for Adults with Major Trauma"

https://pubmed.ncbi.nlm.nih.gov/22511688/

1. The leading cause of death and disability in the young adult population is:
    a. Overdose
    b. Suicide
    c. Trauma
    d. Congenital defects

2. The largest collection of trauma statistics can be found in:
    a. International Trauma Database
    b. National Trauma Data Bank
    c. International Trauma Surgeons Archives
    d. CDC Trauma Indexing

3. According to this study, the number needed to treat for patients transported by HEMS to a level I trauma center is:
    a. 74
    b. 69
    c. 65
    d. 60

4. Limitations to this study include all of the following except:
    a. Missing data points in trauma database
    b. Use of a convenience sample that may not be generalizable
    c. Unknown crew configurations
    d. Known distance traveled by transport crew to reach destination facility

5. Future investigations should attempt to determine how HEMS may lead to improved outcomes, by studying all the the following except:
    a. Patient socioeconomic variables
    b. Treatment rendered prehospitally
    c. Distance traveled to receiving facility
    d. Crew configuration

Appendix J: EMS and Information Sharing Worksheet

Prehospital Emergency Medicine Elective
Worksheet for “EMS and Information Sharing”
https://www.annemergmed.com/article/S0196-0644(14)00524-1/fulltext

1. An Electronic Medical Record that was shareable between EMS and the hospital could provide all of the following benefits, except:
    a. Follow up on patient outcome
    b. Immediate access of hospital staff to prehospital care record
    c. Sharing of medications and allergies
    d. Providing preferred patient hospital destination

2. Obstacles that prevent shared electronic medical records include all of the following except:
    a. Lack of interest in developing appropriate software
    b. Incompatible software
    c. HIPPA regulations
    d. Medical record security

3. According to one study, approximately how much data provided verbally to ER staff is retained?
    a. 15%
    b. 25%
    c. 50%
    d. 75%

4. Reasons why EMS providers are in support of electronic prehospital care reports include all of the following except:
    a. Improved billing and reimbursement rates
    b. Cutting costs by reducing paper documentation
    c. Contributing data to national databases
    d. Improved quality assurance/quality improvement

5. Complete the sentence: “… A hospital may share patient health outcome information…”

    ____

    ____

    ____

Prehospital Emergency Medicine Elective

Worksheet for "Commentary: If we shoot ourselves in the foot, will EMS be there to respond?"

https://pubmed.ncbi.nlm.nih.gov/23178019/

1. Which of the following created the Department of Transportation, and granted it oversight of Emergency Medical Services?
    a. Accidental Death and Disability: The Neglected Disease of Modern Society
    b. Highway Safety Act of 1966
    c. EMS Leadership Act of 1970
    d. EMS Act of 1973

2. Which of the following provided funding to communities, in hopes of establishing regional EMS systems that would eventually be self-sustainable?
    a. Accidental Death and Disability: The Neglected Disease of Modern Society
    b. Highway Safety Act of 1966
    c. EMS Leadership Act of 1970
    d. EMS Act of 1973

3. Reasons for poor financial stability for EMS systems include all of the following except:
    a. Corruption within fund allocations to EMS agencies
    b. Sending resources to evaluate patients who refuse transport, thus not generating cost-of-transport revenue
    c. Low insurance company reimbursement rate compared to actual cost
    d. Increasing cost of employing appropriately trained providers

4. EMS is considered to be at the crossroads of all of the following except:
    a. Medicine
    b. Public Safety
    c. Communication Development
    d. Public Health

5. In the future, EMS could help reduce healthcare costs by:
    a. Refusing to transport patients
    b. Transporting patients to destinations other than Emergency Departments
    c. Decreasing unit staffing levels
    d. Discouraging community paramedicine programs

Prehospital Emergency Medicine Elective

Worksheet for "EMS Makes a Difference: Improved clinical outcomes and downstream healthcare savings"

https://www.annemergmed.com/article/S0196-0644(10)01723-3/fulltext

1. Complete the sentence: "The challenge facing EMS today is to affect…
   ____
   ____
   ____
   ____

2. Which of the following areas has the greatest evidence supporting improved patient outcomes as a result of coordinated prehospital care?
   a. Cerebrovascular disease
   b. Major trauma
   c. Cardiovascular disease
   d. Sepsis

3. EMS interventions that have proven beneficial for patients experiencing a STEMI include all of the following except:
   a. Capture and interpretation of 12 lead EKG
   b. Prehospital activation of cath lab team
   c. Triage of patients to appropriate receiving hospital
   d. Routine use of nitroglycerin for all STEMI patients

4. The Stroke Chain of Survival includes all of the following except:
   a. Early advanced care
   b. Activation of 911 system
   c. Prenotification to receiving facility
   d. Priority transport

5. In the future, paramedics are likely to experience an increased scope of practice in:
   a. Basic wound suturing
   b. In-home care provided to developmentally challenged patients
   c. Not using a glucometer in the treatment of hypoglycemia
   d. Sepsis notification

Appendix M: Case Presentation Checklist

Prehospital Emergency Medicine Elective
Case Presentation Checklist

Student Name: ____

- ☐ Chief Complaint
- ☐ EMS appropriate HPI
- ☐ EMS appropriate PE
- ☐ EMS appropriate treatment
- ☐ Displays and discusses NYS Collaborative Protocol that was used
- ☐ Discusses initial ER workup/management
- ☐ Discusses a challenged faced by the EMS crew
- ☐ Discusses an opportunity for improved communication (EMS to EMS, EMS-to-ED, patient-to-EMS)
- ☐ References and briefly describes at least one scholarly article on a prehospital issue related to the case

Comments:

____

____

____

____

____

____

____

Appendix N: Post-Elective Survey

Prehospital Emergency Medicine Elective Post-Course Survey

Please complete the following survey to the best of your ability. Your answers are anonymous.

Is there anything that stood out in your memory about the elective (ex an experience, an interaction)?

If you were asked to give a lecture to a group of EMTs or paramedics, what topics would you speak about?

If a paramedic were to give a lecture to a group of medical students, what topics would you like to hear about?

Please circle the most correct answer for each of the following questions:

1. In NY, the highest level of 911 prehospital care is provided by:
    a) EMT-Basic
    b) EMT-Paramedic
    c) EMT-Critical Care
    d) EMT-Intermediate

2. In NY, EMT-Basics are allowed to administer all of the following medications except:
    a) Epinephrine
    b) Nitroglycerine
    c) Morphine
    d) Albuterol

3. In NY, EMT-Basics are allowed to perform all of the following skills except:
    a) Spinal motion restriction
    b) IV insertion
    c) Blood glucometry
    d) Semi-automated Defibrillation

4. In Suffolk County, Paramedics are allowed to administer all of the following medications except:
    a) Cardizem
    b) Succinylcholine
    c) Versed
    d) Propofol

5. In Suffolk County, Paramedics are allowed to perform all of the following skills except:
    a) Pericardiocentesis
    b) Needle thoracostomy
    c) Needle cricothyroidotomy
    d) Synchronized cardioversion

6. Prehospital care providers work under the medical license of:
    a) their supervisor
    b) their medical director
    c) the patient's primary care physician
    d) the physician receiving the patient at the hospital

7. In New York, a paramedic's initial education is at least how many hours:
    a) 150
    b) 300
    c) 500
    d) 1000

8. In New York, an EMT-Basic's initial education is at least how many hours:
    a) 150
    b) 300
    c) 500
    d) 1000

9. When a paramedic provides an intervention to a patient without calling a physician, they are using:
    a) Online medical control
    b) Scope of practice
    c) Standing orders
    d) Medical objectives

10. When a paramedic is on scene and wants to perform an intervention that is not specifically mentioned in their protocols, they must:
    a) Call their medical director
    b) Call medical control
    c) Call their supervisor
    d) Call their patient's primary care physician

Appendix O: Open Book Protocol Exam Answers

Prehospital Emergency Medicine Elective
Open Book Protocol Quiz
Reference: NYS Dept of Health Bureau of EMS
Collaborative Advanced Life Support Adult & Pediatric Care Protocols
https://www.health.ny.gov/professionals/ems/pdf/ny_collaborative_protocols_v23.1.pdf

1. Which of the following is NOT a safe way to restrain a child when being transported in an ambulance:
    a. The child's own safety seat, if it is intact
    b. Integrated child restraints that are within the captain's chair/airway seat.
    c. A correctly sized child safety seat that is stocked on the ambulance.
    d. In the arms or lap of the child's parent with the parent properly restrained on the ambulance stretcher.

Answer: A. In general, child safety seats are only designed to absorb one impact; most car insurance companies will cover the cost of child safety seat replacement after a motor vehicle collision. Parents should be encouraged to cut the seat belts within the car seat if placing it out on a curb for garbage collection to deter a passer-by from picking it up and using it themselves.

2. After appropriate on-scene evaluation and stabilization, patients should be transported to:
    a. The closest hospital, regardless of its capabilities
    b. The hospital the patient was born at
    c. The hospital affiliated with the ambulance company
    d. The closest appropriate hospital

Answer: D. Patients should be transported to the closest appropriate receiving hospital, which may not be the nearest hospital. Depending on the patient scenario, the nearest hospital may need to be bypassed to go to one that is, for example, a comprehensive stroke center, burn center, trauma center, or STEMI center.

3. When an advanced airway adjunct (endotracheal tube, supraglottic airway device) is placed, what is required to be utilized to confirm the correct location of the device?
    a. Continuous waveform end-tidal capnography
    b. Pulse oximetry
    c. Lung sounds
    d. 3 lead cardiac monitoring

Answer: A. Continuous waveform end-tidal capnography is required. This means that not only is the capnography being measured numerically, it also has a waveform associated with it. It must be continuous as to detect if the tube migrates, or moves, during patient care or transport.

Pulse oximetry should be used, however there is a several minute lag time between an endotracheal tube being shifted to an incorrect location and when the pulse oximetry will begin to drop. Lung sounds are not a reliable indicator for confirming advanced airway placement, especially in loud environments. Cardiac monitoring is an important monitoring modality in patients, however it is not able to tell an EMS clinician the location of the advanced airway.

4. EMS providers may honor all of the following except:
    a. MOLST form
    b. Non-hospital DNR
    c. DNR bracelet
    d. Hospital DNR

Answer: D. When discussing end-of-life care and patient wishes, it is crucial to document patient's wishes on a form that can be honored by EMS. This includes a non-hospital DNR, or MOLST form. DNR bracelets or necklaces may also be honored by EMS. In the event that the physical MOLST or DNR is unable to be located, or a hospital DNR is present, the EMS crew can call medical direction for pronouncement orders in accordance with the patient's wishes. As physicians, it's important that we emphasize that these documents be readily available (some patients tape them on the wall above their bed, or hang on their refrigerator), and that family or caregivers are aware of their location.

5. For patients in anaphylactic shock, paramedics may administer all of the following medications under standing order EXCEPT:
    a. Epinephrine 0.3mg IM
    b. Epinephrine infusion
    c. Diphenhydramine 50mg IV or IM
    d. Dexamethasone 10mg PO, IM, or IV

Answer: B. Contact with medical control is required before starting an epinephrine infusion.

6. For agitated patients who are extremely combative and at immediate risk of causing physical harm to themselves or others, paramedics may perform all of the following interventions under standing order EXCEPT:
    a. Midazolam 5mg IM or IV; may repeat up to 10mg
    b. Ketamine 250mg IM
    c. Haloperidol 5mg IV or IM
    d. Application of soft restraints

Answer: C. Paramedics must contact medical control for approval to administer haloperidol.

7. For adult patients with symptomatic bradycardia, paramedics may perform all of the following interventions EXCEPT:
    a. Transcutaneous pacing
    b. Norepinephrine infusion
    c. Atropine 1mg IV
    d. Synchronized cardioversion

Answer: D. Synchronized cardioversion is an intervention to be used in a fast, unstable rhythm. This skill is able to be performed by paramedics without physician permission under the correct clinical circumstances.

8. For patients experiencing a STEMI, what intervention can a paramedic perform that an EMT can not?
    a. Administer a bolus of IV fluids if the patient becomes hypotensive
    b. Administer ASA 324mg
    c. Transmit a 12-lead EKG
    d. Assist a patient to take their prescribed nitroglycerin if their systolic BP is >120mmHg

Answer: A. An EMT is not allowed to start IVs or administer fluids; EMTs are able to perform all other options.

9. In adult patients with pulmonary edema and shortness of breath, EMTs can offer which of the following interventions, if trained and equipped:
    a. Continuous Positive Airway Pressure (CPAP)
    b. Furosemide 40mg IVP
    c. Albuterol 2.5mg in 3mL nebulizer
    d. IV placement

Answer: A. While an EMT is able to administer albuterol nebulizers, that would not be indicated in pulmonary edema. IV placement and IV medications are not within the EMT scope of practice.

10. For pediatric patients with severe asthma or wheezing, which of the following interventions requires medical control approval for a paramedic to perform:
    a. Epinephrine 0.01mg/kg IM
    b. Dexamethasone 10mg PO, IM, or IV
    c. Albuterol and ipratropium nebulizers to a total of three doses
    d. Epinephrine infusion

Answer: D. An epinephrine infusion would require physician approval.

11. In adult patients with hyperkalemia in cardiac arrest, what medication is not available for paramedics to administer?
    a. Sodium bicarbonate
    b. IV fluid bolus
    c. Insulin
    d. Calcium chloride

Answer: C. Insulin is not carried prehospitally in New York State. This is a medication that a patient would receive in the Emergency Department to shift the potassium intracellularly.

12. What intervention can a paramedic perform for a patient with a blood glucose reading greater than 400 mg/dL?
    a. Insulin 10 units
    b. Normal saline 500mg IV bolus
    c. Dextrose 10%, 25 grams
    d. Glucagon 1mg IM

Answer: B. Hyperglycemia is treated prehospitally with IV fluids presuming there are no clinical symptoms of pulmonary edema or heart failure. Glucagon and Dextrose can be given for patients with hypoglycemia.

13. For adult patients experiencing pain, all of the following medications can be administered by a paramedic without contacting a physician EXCEPT:
    a. Acetaminophen
    b. Fentanyl
    c. Ketorolac
    d. Ketamine

Answer: D. Pain dose ketamine requires medical control authorization to use.

14. Which of the interventions is crucial for a paramedic to perform specifically if a patient who is seizing is believed to be pregnant?
    a. Magnesium 4g IV over 20 minutes
    b. Obtain a blood glucose
    c. Midazolam 5mg IV/IM/IN
    d. A second dose of midazolam 5mg IV/IM/IN

Answer: A. While all the above are appropriate interventions for a patient who is seizing, magnesium must be given in the case of an eclamptic seizure.

15. For patients with a suspected stroke, an EMT should perform all of the following interventions except:
    a. A blood glucose level
    b. A Cincinnati Stroke Scale
    c. A hospital prenotification
    d. Administer metoprolol for hypertension

Answer: D. Blood pressure management in the setting of suspected stroke is limited to a medical control option for paramedics.

16. Reasonable care of an amputated extremities includes all of the following except:
    a. Wrapping with sterile dressing moistened with sterile saline
    b. Placing directly on ice
    c. Placing in a water-tight container
    d. Reducing any additional trauma to the tissue

Answer: B. The limb should not be placed directly on ice; it should be wrapped with moist gauze, placed in a plastic bag or other airtight container, and then indirectly placed on ice or cold packs, taking care to avoid freezing the tissue.

17. In the setting of unconditional bleeding of an extremity, the most optimal placement of a tourniquet is:
    a. 2-3 inches proximal to the wound
    b. 2-3 inches distal to the wound
    c. On the wound as to act as a pressure dressing
    d. As close to the torso as possible

Answer: A. Ideally, the tourniquet is placed 2-3 inches proximal to the wound. If one tourniquet is unable to control the bleeding, a second, more proximal tourniquet may be applied.

18. In the setting of an adult patient with chest trauma with concern for tension pneumothorax who is not in cardiac arrest, which level provider is able to perform a needle decompression of the chest under standing orders?
    a. EMT
    b. EMT-CC
    c. Paramedic
    d. Advanced

Answer: C. Only a paramedic is able to perform a needle decompression for a suspected tension pneumothorax for a patient who is not in cardiac arrest under standing order. If the patient is in cardiac arrest, Advanced and CC can perform a needle decompression under standing order. Needle decompression is not within the EMT scope of practice.

19. When performing an RMA, Medical Control should be contacted if any “RMA High Risk Criteria” are present. These criteria include all of the following except:
    a. Age >65
    b. Pulse >140
    c. Serious chief complaint or significant mechanism of injury
    d. Fever in a newborn or infant less than 8 weeks old

Answer: B. A patient with a heart rate > 120 or <50 is concerning and should be considered a high risk scenario.

20. Paramedics may obtain vascular access via all of these routes except:
    a. Brachiocephalic intravenous (IV)
    b. Intraosseous (IO)
    c. Internal jugular vein (IJ)
    d. External jugular vein (EJ)

Answer: C. If properly trained, paramedics can access in-place central lines such as a PICC, however it is not within paramedic scope of practice to directly obtain vascular access to the internal jugular vein. The external jugular vein is considered a peripheral vein, and therefore is allowed to be directly accessed by a paramedic.

Appendix P: Scholarly Article Worksheet Answers

Prehospital Emergency Medicine Elective
Answers for Comprehension Quizzes

REQUIRED:
"Optimizing the Patient Handoff Between Emergency Medical Services and the Emergency Department"
1. C
2. D
3. A
4. D
5. C

Pick 2 of the following 4:
"EMS and Information Sharing"
1. D
2. A
3. C
4. B
5. "… a hospital may share patient health outcome information with the EMS provider for quality improvement as long as both entities have or had a relationship with the patient in question."

"EMS Makes a Difference: Improved clinical outcomes and downstream healthcare savings"
1. "The challenge facing EMS today is to affect a system-wide transformation from practices based on tradition and expert opinion to adoption of national guidelines and protocols that have been developed through a rigorous examination of the scientific evidence and a systematic guideline development process."
2. C (p. 3)
3. D (p. 3)
4. A (p. 6)
5. A (p. 13)

"**Association Between Helicopter vs Ground Emergency Medical Services and Survival for Adults with Major Trauma"**
1. C
2. B
3. C
4. D
5. A

"Commentary: If we shoot ourselves in the foot, will EMS be there to respond?"
1. B
2. D
3. A

Appendix P: Scholarly Article Worksheet Answers

4. C
5. B
